# National, regional and provincial prevalence of carotid atherosclerosis and carotid plaque in Chinese adults: an updated systematic review and modelling analysis

**DOI:** 10.7189/jogh.16.04089

**Published:** 2026-02-27

**Authors:** Shiyi Shan, Jing Wu, Jiali Zhou, Liying Zhou, Meijia Xu, Longzhu Zhu, Igor Rudan, Peige Song, Global Health Epidemiology Research Group (GHERG)

**Affiliations:** 1 Center of Clinical Big Data and Analytics of the Second Affiliated Hospital and School of Public Health, Zhejiang University School of Medicine, Hangzhou, China; 2 Sir Run Run Shaw Hospital, Medical College of Zhejiang University Hangzhou, Zhejiang, China; 3 Centre for Global Health, Usher Institute, University of Edinburgh, Edinburgh, Scotland, UK; 4 Nuffield Department of Primary Care Health Sciences, Oxford University, Oxford, UK; 5 Zhejiang Key Laboratory of Intelligent Preventive Medicine, Hangzhou, Zhejiang, China

## Abstract

**Background:**

Carotid atherosclerosis (CAS), a major contributor to cardiovascular morbidity in China, exhibits substantial geographic disparities that remain unquantified at the provincial level. We aimed to provide updated national, regional, and provincial prevalence of CAS or carotid plaque (CP) among Chinese adults aged 30–79 years.

**Methods:**

We searched CNKI, Wanfang, China Science and Technology Journal Database (VIP), PubMed, Embase, and MEDLINE for articles published after 2010 that reported on the prevalence of CAS or CP in the general Chinese population. We used a multilevel mixed-effects meta-regression model to estimate the national age- and sex-specific prevalence of CAS and CP. We assessed the relationships of associated factors with CAS and CP using random-effects meta-analysis. The pooled odds ratios were then incorporated into ‘associated factor-based’ model, alongside provincial-level associated factor data, to estimate national case numbers across provinces.

**Results:**

Based on data from 82 articles, we estimated the overall prevalences of CAS and CP to be 36.35% (95% confidence interval (CI) = 29.74–43.44) and 26.27% (95% CI = 23.17–29.59), respectively. We noted associations between high prevalence and advancing age, with males having a higher prevalence across the whole age spectrum compared to females. A total of 320.25 million (95% CI = 262.03–382.72) and 231.48 million (95% CI = 204.19–260.70) population aged 30–79 years were affected by CAS and CP in mainland China. Current smoking, hypertension, and diabetes were associated with CAS and CP, with dyslipidaemia only associated with CP. Northeast China recorded the highest prevalence of CAS (39.31%; 95% CI = 32.25–46.81) and CP (28.56%; 95% CI = 25.21–32.13) in 2020. Sichuan Province had the highest prevalence of both CAS (42.14%; 95% CI = 34.79–49.92) and CP (30.77%; 95% CI = 27.26–34.50), while Tibet showed the lowest (CAS: 26.80%; 95% CI = 21.35–32.92; CP: 18.74%; 95% CI = 16.33–21.39).

**Conclusions:**

CAS and CP are highly prevalent in China, with substantial age trends, sex differences, and regional disparities. Targeted prevention strategies and evidence-based resource allocation are needed to manage the high burden of CAS and CP and their associated factors.

**Registration:**

PROSPERO: CRD420251033132.

Cardiovascular disease (CVD) remains the leading cause of mortality worldwide, with a growing burden in low-income and middle-income countries, including China [1]. One pathological process underlying most cardiovascular and cerebrovascular events is atherosclerosis, a chronic, progressive condition characterised by lipid accumulation and fibrotic changes in the arterial wall [2]. The disease often begins early in life and can remain clinically asymptomatic for decades before progressing to advanced lesions such as plaque, stenosis, or occlusion, which may precipitate acute events, including ischaemic stroke or myocardial infarction [3].

Carotid arteries are particularly vulnerable to atherosclerotic changes due to their anatomical bifurcations and haemodynamic characteristics [4]. Thus, the carotid system has been a focus for early detection of subclinical atherosclerosis, particularly in asymptomatic populations [5]. Carotid intima-media thickness (cIMT), assessed non-invasively *via* ultrasonography, is a well-established surrogate marker of early atherosclerosis [6,7]. Elevated cIMT has been associated with increased risk of further cardiovascular events, including stroke and coronary heart disease, and may reflect systemic atherosclerotic burden across multiple vascular beds [8–11].

Despite its clinical importance, population-level data on carotid atherosclerosis (CAS) remain limited in many settings, especially in large, heterogeneous countries such as China. Disparities in diagnostic definitions, imaging protocols, and access to standardised care complicate efforts to generate reliable estimates of disease prevalence. Previous epidemiological studies have indicated substantial geographic variation in CAS prevalence across China. For instance, a cross-sectional study by Fu *et al*. revealed that the prevalence of CAS was the highest in the country’s north region [12]. However, most existing estimates were derived from health check-up populations, which may not adequately reflect the general population due to selection bias related to health awareness and socioeconomic status.

In 2018, we performed a systematic review and meta-analysis to estimate the prevalence of CAS and carotid plaque (CP) in China, but had insufficient data to explore variations at the provincial level [13]. Over the past decade, China has undergone rapid demographic and epidemiological transitions, characterised by population ageing and an increasing prevalence of hypertension, diabetes, obesity, and other atherosclerotic risk factors [14]. These changes underscore the need for updated and more granular epidemiological assessments to inform policy and guide prevention strategies.

## METHODS

We registered the protocol for this study at PROSPERO (CRD420251033132), and conducted and reported its findings in accordance with the PRISMA guidelines [15] and the GATHER statement [16].

### Search strategy and selection criteria

We have previously estimated the prevalence of CAS and CP in China through a systematic review and meta-analysis published in 2018 [13]. In the present study, we aimed to provide the most up-to-date and comprehensive estimates of CAS and CP prevalence in the general Chinese population. We systematically searched three Chinese (CNKI, Wanfang, China Science and Technology Journal Database) and three global bibliographic databases (PubMed, Embase, and MEDLINE) for articles published between 16 April 2017 (the end of the search period for our previous review [13]) and 12 August 2024. The search strategy (Appendix S1 in the **Online Supplementary Document**) combined MeSH terms and free-text keywords related to carotid atherosclerosis, carotid plaque, and prevalence (in addition to ‘China’ in the three global databases), without any language restrictions.

After deduplication, two researchers (MX, LZ) independently screened the titles and abstracts of all records, followed by the full-text review of any that were potentially eligible. We included population-based studies conducted in the general population in China, provided they used ultrasound imaging to assess CAS or CP, reported numerical prevalence estimates, defined CAS and CP in line with the 2016 European Guidelines on Cardiovascular Disease Prevention in Clinical Practice [5] and the Mannheim Carotid Intima-Media Thickness and Plaque Consensus [6]; and conducted in or after 2010 (to ensure consistency in diagnostic standards). Specifically, CAS had to be defined as a cIMT ≥1.0 mm, and CP as either a focal structure that encroaches into the arterial lumen of ≥0.5 mm or ≥50% thicker than the surrounding wall; or a cIMT ≥1.5 mm [5,6].

We excluded studies conducted in hospital-based or clinical samples, reviews, conference abstracts, commentaries, case reports, viewpoints, and letters. In cases where multiple publications reported on the same study population, we retained the most recent or most comprehensive article with the largest sample size. We also included 18 additional articles from our 2018 review that were conducted in or after 2010 [13]. Lastly, we manually screened the reference lists of included studies and relevant reviews to identify additional eligible articles.

### Data extraction and quality assessment

Using a standardised data collection template, two researchers (MX, LZ) independently extracted the following data from the included articles, with checks by a third researcher (SS): bibliographic information (title, authors, publication year); study characteristics (location, study year, setting (urban/rural/mixed), inclusion and exclusion criteria, sample size), participant demographics (age range, mean or median age, the proportion of female participants), prevalence data (sample size and the number of people with CAS or CP, stratified by age and sex, where available).

Where study years were not reported, we imputed the year as three years prior to the publication year, based on the average lag observed across included studies (Appendix S3 and Table S1 in the **Online Supplementary Document**). When age groups were right-censored (*e.g.* 80 years and above), we employed two methods to impute the missing age band: if no age statistics were reported, we assumed the same age-band width as other age groups within the same study; if the average or median age was available, we defined the missing age band by centring it around the reported statistic. For missing data on the proportion of female participants, we assumed a value of 0.50. A subset of articles additionally investigated associations of potential associated factors with CAS or CP. We included only those that reported adjusted odds ratios (aORs) with 95% confidence intervals (CIs) from multivariable analyses, with consistent definitions and uncertainty estimates.

We assessed the quality of studies using the Quality Assessment Tool [17], which evaluates the overall risk of bias across five dimensions: sample population, sample size, participation rate, outcome assessment, and analytical methods. Each dimension is scored from zero (low) to two points (high), with a total score ranging from zero to ten (Appendix S3 and Table S2 in the **Online Supplementary Document**).

Disagreements during screening, data extraction, and quality assessment were resolved through discussion or by consultations with a senior researcher (PS).

### Statistical analysis

**Figure 1:**
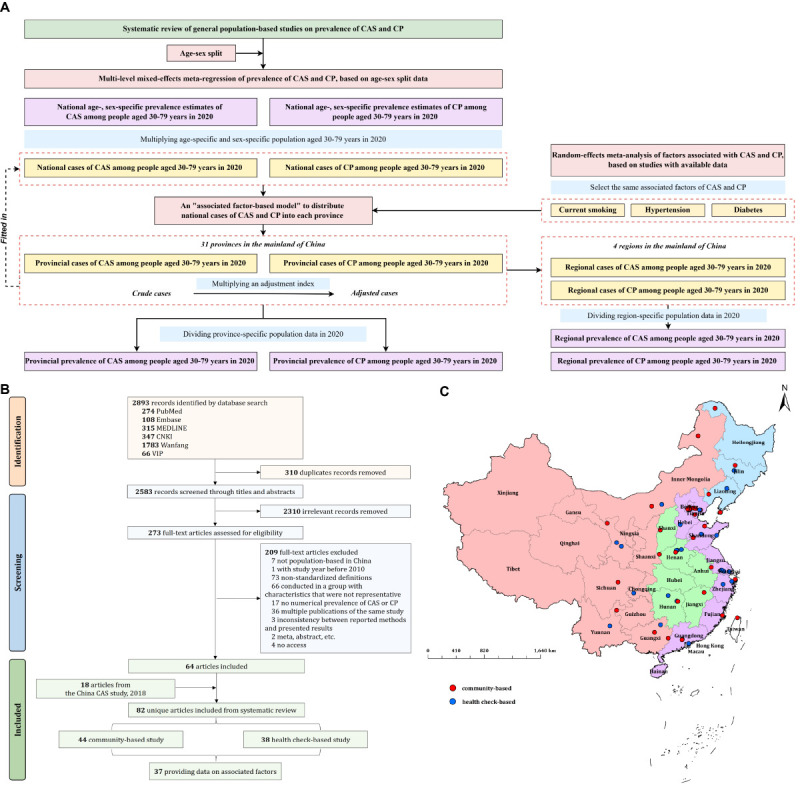
**Panel A.** Study framework. **Panel B.** Systematic review flow diagram. **Panel C.** Location of included studies. Five articles with multiple study locations are not shown in the map. CAS – carotid atherosclerosis, CP – carotid plaque.

The overall framework of the statistical analysis is summarised in **Figure 1**, Panel A, with further methodological details provided in Appendix S2 in the **Online Supplementary Document**.

#### Age and sex splitting of prevalence data

We employed an age-sex-splitting procedure to improve the availability and comparability of data for prevalence modelling. This approach enabled the transformation of aggregated prevalence data into age-specific and sex-specific estimates. We used multilevel mixed-effects meta-regression models to assess the association of age and sex with CAS and CP prevalence, and to generate continuous age- and sex-specific prevalence curves. Prevalence estimates were logit-transformed to stabilise variance for proportions approaching 0 or 1. For studies reporting zero cases, a small nonzero value (0.0005) was substituted to permit logit transformation. We specified the model as:



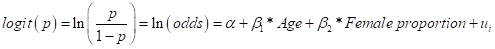



Therefore,



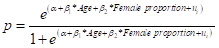



Here, *p* denotes prevalence, *α* is the intercept term, *β* is the coefficient, and *u_i_* represents the random effects at the study and province level, accounting for within-study clustering.

We used the age- and sex-specific prevalence curves of CAS and CP as ‘age & sex’ patterns to disaggregate combined-sex data into sex-specific estimates. Likewise, we split broad age categories into one-year age intervals using the fitted age-specific pattern (Table S3 and Figure S1 in the **Online Supplementary Document**).

#### Epidemiological modelling of national prevalence for CAS and CP

Using the community-based age-sex-split prevalence data for CAS and CP, we examined the age- and sex-adjusted effects of key cluster-level variables, including study year and economic region, on CAS and CP prevalence using multivariable meta-regression (Table S4 in the **Online Supplementary Document**). Neither covariate was found to be significantly associated with prevalence and thus was not retained (Table S5 in the **Online Supplementary Document**). Therefore, the final national level model only incorporated age and sex as fixed effects and random intercepts for study and province:



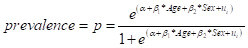



Analyses were restricted to individuals aged 30–79 years, consistent with available datapoints and prior literature [13]. We then multiplied age- and sex-specific prevalence estimates by the corresponding population data from the 2020 census in China [18] to derive the number of individuals aged 30–79 years with CAS and CP in mainland China in 2020 (national case envelopes).

#### Epidemiological modelling of regional and provincial prevalence for CAS and CP

A subset of studies additionally investigated associated factors of CAS and CP using multivariable analysis with similar definitions. We performed a random-effects (restricted maximum likelihood) meta-analysis to pool ORs for associated factors. As a rule, at least three independent datapoints were required for each associated factor to ensure robustness. Based on findings of, and for consistency with, previous literature, we selected three commonly reported associated factors, namely current smoking, hypertension, and diabetes, for further modelling (Table S6 and Figure S2 in the **Online Supplementary Document**)[19].

We used an ‘associated factor-based’ model to allocate national case envelopes to 31 provinces in mainland China, obtaining the national and provincial prevalence of these three associated factors in China from previous large-scale studies [20-23]. We estimated provincial case numbers for CAS and CP as a function of local associated factor prevalence and population size.



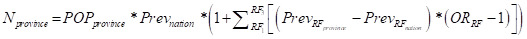



Here, *N_province_* and *POP_province_* are the number of CAS or CP cases and population size aged 30–79 years in each province or municipality, *Prev_nation_* indicates the estimated national prevalence of CAS or CP, *RF_1_* to *RF_3_* are the three selected associated factors, namely current smoking, hypertension, and diabetes, *Prev_province_* and *Prev_RFnation_* are the prevalence of the three associated factors in each province or municipality and the mainland of China, and *OR_RF_* is the synthesised OR of current smoking, hypertension, and diabetes.

Lastly, we calculated the provincial prevalence by dividing estimated case counts by the corresponding provincial population. We grouped provinces into four economic regions (East, Central, West, and Northeast China), according to official classifications (Table S7 in the **Online Supplementary Document**). We derived regional case numbers by summing provincial estimates, and calculated regional prevalences by dividing total case numbers by the regional population.

We performed all statistical analyses in *R*, version 4.4.2 (R Core Team, Vienna, Austria). Maps were produced using ArcMap, version 10.8 (Environmental Systems Research Institute, Redlands, CA).

## RESULTS

### Systematic review and study characteristics

We retrieved 2893 records from our database search (**Figure 1**, Panel), with 2583 remaining after deduplication and 273 after the initial title/abstract screening. Of these, 64 articles met the inclusion criteria after the full-text review, with an additional 18 added from our previous study [13] for total of 82 articles encompassing 11 111 495 participants (**Figure 1**, Panel C; Tables S8–10 and Appendix S4 in the **Online Supplementary Document**). Among them, 47 (57.32%) reported the prevalence of CAS, and 65 (79.27%) reported the prevalence of CP, nearly half (n = 40, 48.78%) were published after 2019, and just above half (n = 44, 53.66%) were based on community-dwelling populations. Most of the raticles (n = 46, 56.10%) were given a quality score of six or higher.

### National prevalence and case number of CAS and CP

**Figure 2:**
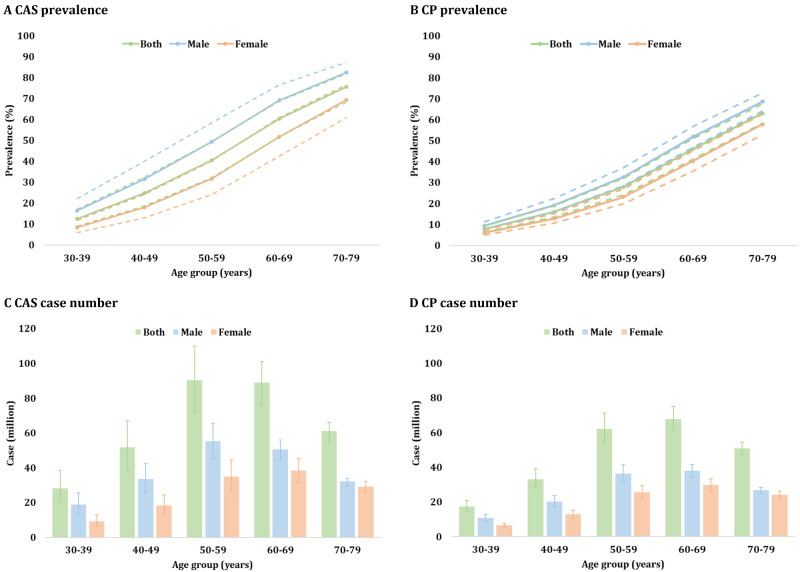
National prevalence and case number of carotid atherosclerosis and carotid plaque among individuals aged 30–79 years in China. **Panel A.** National sex-specific prevalence of CAS by age group. **Panel B.** National sex-specific prevalence of CP by age group. **Panel C.** National sex-specific case number of CAS by age group. **Panel D.** National sex-specific case number of CP by age group. CAS – carotid atherosclerosis, CP – carotid plaque.

The overall national prevalence of CAS among individuals aged 30–79 years was 36.35% (95% CI = 29.74–43.44), corresponding to 320.25 million (95% CI = 262.03–382.72) affected individuals in 2020 (**Figure 2**, **Table 1**). Prevalence increased progressively with age, with males consistently exhibiting higher prevalence than females. In males, the overall prevalence of CAS was 42.80% (95% CI = 35.63–50.27), ranging from 16.42% (95% CI = 11.91–22.21) in individuals aged 30–39 years to 82.54% (95% CI = 76.48–87.30) in those aged 70–79 years. In females, the overall prevalence was 29.77% (95% CI = 23.73–36.47), ranging from 8.50% (95% CI = 6.00–11.91) in individuals aged 30–39 years to 69.28% (95% CI = 60.85–76.61) in those aged 70–79 years. Among the 320.25 million cases, 59.43% were males (190.34 million; 95% CI = 158.44–223.56) and 40.57% were females (129.91 million; 95% CI = 103.59–159.16). We observed a distinct age-specific distribution pattern of case numbers, with the 50–59-year age group having the largest estimated case count at 90.29 million (95% CI = 71.74–110.06).

**Table 1:** Estimated sex-specific prevalence and case number of carotid atherosclerosis and carotid plaque in the mainland of China in 2020, by age group

Age in years	Prevalence in % (95% CI)			Case number in millions (95% CI)		
**Both**	**Male**	**Female**	**Both**	**Male**	**Female**
**CAS**						
30–39	12.58 (9.04–17.21)	16.42 (11.91–22.21)	8.50 (6.00–11.91)	28.07 (20.17–38.40)	18.85 (13.67–25.50)	9.21 (6.50–12.90)
40–49	24.95 (18.75–32.33)	31.62 (24.16–40.15)	18.00 (13.11–24.17)	51.70 (38.86–66.99)	33.46 (25.56–42.49)	18.24 (13.29–24.50)
50–59	40.57 (32.24–49.45)	49.36 (40.18–58.58)	31.67 (24.20–40.22)	90.29 (71.74–110.06)	55.24 (44.97–65.56)	35.04 (26.77–44.50)
60–69	60.41 (51.56–68.65)	69.20 (60.75–76.54)	51.74 (42.49–60.87)	89.04 (75.99–101.18)	50.66 (44.47–56.03)	38.38 (31.52–45.15)
70–79	75.66 (68.38–81.76)	82.54 (76.48–87.30)	69.28 (60.85–76.61)	61.16 (55.27–66.08)	32.12 (29.76–33.97)	29.04 (25.50–32.11)
30–79	36.35 (29.74–43.44)	42.80 (35.63–50.27)	29.77 (23.73–36.47)	320.25 (262.03–382.72)	190.34 (158.44–223.56)	129.91 (103.59–159.16)
**CP**						
30–39	7.79 (6.47–9.34)	9.42 (7.85–11.26)	6.06 (5.02–7.30)	17.38 (14.45–20.83)	10.81 (9.01–12.93)	6.56 (5.43–7.90)
40–49	16.04 (13.56–18.87)	19.13 (16.24–22.39)	12.81 (10.75–15.20)	33.23 (28.09–39.10)	20.24 (17.19–23.69)	12.99 (10.90–15.41)
50–59	27.90 (24.13–32.01)	32.61 (28.41–37.10)	23.15 (19.80–26.86)	62.11 (53.71–71.24)	36.50 (31.80–41.52)	25.61 (21.91–29.72)
60–69	46.00 (41.20–50.87)	51.84 (46.90–56.75)	40.23 (35.58–45.06)	67.80 (60.73–74.97)	37.96 (34.34–41.55)	29.84 (26.39–33.43)
70–79	63.06 (58.41–67.49)	68.67 (64.26–72.77)	57.85 (52.97–62.58)	50.97 (47.21–54.55)	26.72 (25.01–28.32)	24.25 (22.20–26.23)
30–79	26.27 (23.17–29.59)	29.74 (26.39–33.28)	22.74 (19.90–25.82)	231.48 (204.19–260.70)	132.23 (117.35–148.01)	99.25 (86.83–112.69)

CAS – carotid atherosclerosis, CI – confidence interval, CP – carotid plaque

For CP, the national prevalence among adults aged 30–79 years was 26.27% (95% CI = 23.17–29.59), equating to 231.48 million individuals (95% CI = 204.19–260.70) (**Figure 2**, **Table 1**). The prevalences of CP for males and females aged 30–79 years were 29.74% (95% CI = 26.39–33.28) and 22.74% (95% CI = 19.90–25.82), respectively. In males, the prevalence ranged from 9.42% (95% CI = 7.85–11.26) for 30–39 years to 68.67% (95% CI = 64.26–72.77) for 70–79 years. In females, the prevalence was slightly lower than in males across the whole age spectrum of 30–79 years, ranging from 6.06% (95% CI = 5.02–7.30) to 57.85% (95% CI = 52.97–62.58). Of the total cases, 57.12% were males (132.23 million; 95% CI = 117.35–148.01) while 42.88% were females (99.25 million; 95% CI = 86.83–112.69). The 60–69-year age group accounted for the highest case number, with a total of 67.80 million (95% CI = 60.73–74.97).

### Associated factors for CAS and CP

Several factors were consistently associated with increased prevalence of both CAS and CP (Figure S2 and Table S7 in the **Online Supplementary Document**). For CAS, advanced age was associated with higher odds of CAS, with a pooled OR of 1.10 (95% CI = 1.07–1.13) per year increase. Being male was associated with a significantly elevated odds of having CAS compared with being female (meta-OR = 1.80; 95% CI = 1.62–2.01). Current smoking was also associated with higher odds of CAS (meta-OR = 1.53; 95% CI = 1.37–1.71), as were hypertension (meta-OR = 1.58; 95% CI = 1.33–1.89) and diabetes (meta-OR = 1.71; 95% CI = 1.43–2.05).

We observed similar patterns for CP. Age remained a strong associated factor (meta-OR = 1.09; 95% CI = 1.04–1.13), while being male was associated with more than double the odds of CP compared to being female (meta-OR = 2.03; 95% CI = 1.66–2.48). Current smoking (meta-OR = 1.46; 95% CI = 1.22–1.75), hypertension (meta-OR = 1.62; 95% CI = 1.62–1.63), and diabetes (meta-OR = 1.72; 95% CI = 1.48–2.00), and dyslipidaemia (meta-OR = 1.29; 95% CI = 1.01–1.65) were also significantly associated with CP.

### Regional and provincial prevalence and case number of CAS and CP

**Figure 3:**
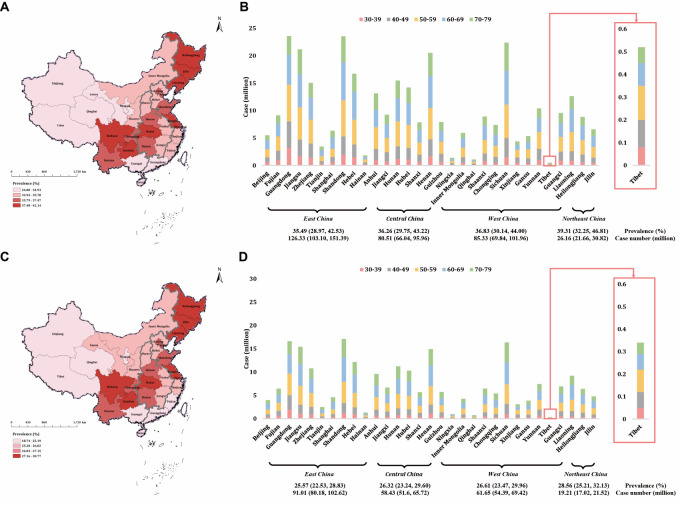
Regional and provincial prevalence and case number of carotid atherosclerosis and carotid plaque among individuals aged 30–79 years in the mainland of China in 2020. **Panel A.** Provincial prevalence of CAS. **Panel B.** Regional and provincial prevalence and case number of CAS. **Panel C.** Provincial prevalence of CP. **Panel D.** Regional and provincial prevalence and case number of CP. CAS – carotid atherosclerosis, CP – carotid plaque.

We observed marked geographical variation in estimated CAS and CP prevalence across China’s four economic regions and 31 provinces (**Figure 3**; Table S11–14 in the **Online Supplementary Document**). In 2020, Northeast China had the highest regional prevalence of CAS (39.31%; 95% CI = 32.25–46.81), while East China had the lowest (35.49%; 95% CI = 28.97–42.53). Despite this, East China accounted for the largest share of national cases, with an estimated 126.33 million (95% CI = 103.10–151.39) affected individuals. At the provincial level, our model estimated that Sichuan had the highest prevalence (42.14%; 95% CI = 34.79–49.92), while Guangdong had the largest estimated number of cases (23.58 million; 95% CI = 18.98–28.66). Tibet, meanwhile, showed both the lowest prevalence (26.80%; 95% CI = 21.35–32.92) and the lowest number of cases (0.51 million; 95% CI = 0.40–0.62).

Similarly for CP, Northeast China again had the highest estimated regional prevalence (28.56%; 95% CI = 25.21–32.13), while East China had the lowest (25.57%; 95% CI = 22.53–28.83), but contributed the greatest number of cases (91.01 million; 95% CI = 80.18–102.62). At the provincial level, Sichuan bore the highest CP prevalence (30.77%; 95% CI = 27.26–34.50), while Tibet had the lowest (18.74%; 95% CI = 16.33–21.39). Shandong had the highest number of CP cases (17.12 million; 95% CI = 15.12–19.25), while Tibet had the lowest (0.35 million; 95% CI = 0.31–0.40).

## DISCUSSION

This systematic review and modelling analysis based on 82 articles covering 11.1 million participants provides the most updated and nationally representative prevalence estimates of CAS and CP among Chinese adults aged 30–79 years at the national, regional and provincial levels. In 2020, the prevalence of CAS and CP was 36.35% (95% CI = 29.74–43.44) and 26.27% (95% CI = 23.17–29.59), corresponding to 320.25 million (95% CI = 262.03–382.72) and 231.48 million (95% CI = 204.19–260.70) affected individuals, respectively. We found advanced age, being male, current smoking, hypertension, and diabetes to be associated with both CAS and CP. We also observed substantial geographical variations, with Northeast China showing higher prevalence, and East China bearing the largest case burden. At the provincial level, Sichuan had the highest prevalence of both CAS (42.14%) and CP (30.77%), Guangdong and Shandong had the largest number of cases for CAS (23.58 million) and CP (17.12 million), and Tibet had the lowest prevalence and case numbers for both CAS (26.80%, 0.51 million) and CP (18.74%, 0.35 million).

Compared with our 2010 national estimates (27.22% for CAS and 20.15% for CP) [13], the higher prevalences in 2020 (36.35% for CAS and 26.27% for CP) suggest a significant increase over the past decade. This trend likely reflects the persistent burden of cardiovascular risk factors in China, including smoking, hypertension, diabetes, and population ageing [20–24]. These prevalence rates also exceed the global estimates for 2015 (27.6% for increased cIMT and 21.1% for CP) [25], potentially due to regional differences in risk factor profiles, population characteristics, and measurement practices [20–24]. While a recent study by Fu *et al*. provided provincial-level estimates of CAS prevalence in China based on health check-up data from the Meinian Healthcare Group [12], important differences in study populations warrant consideration. Participants undergoing private health check-ups typically have higher socioeconomic status and better overall health profiles compared with the general population, which may explain Fu and colleagues’ lower estimates of the prevalence of CAS (26.2%) and CP (21.0%) [12]. In contrast, we synthesised data from community-based epidemiological investigations, aiming to provide more representative estimates for the general adult population across China.

As expected for a progressive vascular condition, the prevalence of CAS and CP increased steadily with advancing age [26]. However, our findings also highlight the early onset of the two diseases; individuals aged 30–39 years had a CAS prevalence of 12.58%, and a CP prevalence of 7.79%, highlighting that atherosclerosis is not specific to older adults [3]. Given that CAS often begins early in life and remains asymptomatic for decades [3,5], these findings emphasise the need for early detection and prevention strategies starting in young adulthood [27].

We noted marked sex differences in the prevalence of CAS and CP, with men showing substantially higher rates than women. This pattern is consistent with both our previous national estimates and global data [13,25], and may be partly attributed to the protective vascular effects of oestrogens before menopause, which support endothelial function and regulate lipid metabolism [28]. Other biological factors, such as sex-specific inflammatory profiles and anatomical differences in carotid artery geometry influencing hemodynamic forces, may also contribute to this disparity [28,29]. Lifestyle factors, such as higher smoking rate greater exposure to hypertension and diabetes among men, likely exacerbate the sex gap in CAS prevalence [20–23]. Although men bear a higher prevalence, the cardiovascular health needs of women should not be underestimated. A European study has shown that women are less likely to receive carotid imaging compared to men, potentially leading to underdiagnosis [30]. Cultural and social norms, where women often prioritise caregiving roles over personal health, may further delay diagnosis and treatment [31]. These findings underscore the need for targeted public health interventions. Among men, efforts should focus on reducing smoking rates and improving management of hypertension and diabetes. For women, ensuring equitable access to preventive screening and early interventions is critical. In both sexes, early detection and timely initiation of preventive strategies will be essential to mitigate the rising burden of CAS.

We further identified current smoking, hypertension, and diabetes were significantly associated with the risk of CAS and CP. These findings have been widely explored in previous investigations [32,33], and align with our previous studies [13,25]. These results reinforce the importance of intensified risk factor control, particularly among high-risk subgroups such as men, smokers, and individuals with existing cardiovascular disease. Dyslipidaemia was further identified as an associated factor of CP, consistent with its known role in atherosclerotic progression [34]. However, due to the limited number of included studies and absence of prevalence data, we did not incorporate dyslipidaemia into the ‘associated factor-based model’ used to distribute provincial estimates. In addition, inconsistent definitions and limited datapoints precluded robust meta-analyses for other potential factors, such as obesity. These gaps highlight the need for more systematic and standardised evaluations of a broader range of associated factors.

Our analysis showed considerable regional and provincial variations in the prevalence of CAS and CP across China. Among the four economic regions, Northeast China had the highest prevalence, whereas East China, despite having lower prevalence rates, carried the largest absolute burden of cases due to its large population size. At the provincial level, Sichuan stood out with the highest prevalence for both CAS and CP, while Shandong accounted for the greatest number of cases. These disparities are likely shaped by a combination of regional differences in exposure to major cardiovascular risk factors, socioeconomic development, urban-rural divide, healthcare access, and lifestyle patterns. In Sichuan, for example, relatively high smoking rates among men and elevated prevalence of hypertension and diabetes may contribute to the higher rates of carotid pathology [20–23]. Similarly, although smoking is moderately prevalent in the Northeast China, the region shows consistently high rates of hypertension and diabetes [21–23]. Dietary habits, particularly high salt intake in northern populations, and variations in health literacy and preventive health service utilisation further compound these differences [20–23].

To our knowledge, this study provides the most comprehensive and up-to-date prevalence estimates of CAS and CP among the general population in China in 2020. Its major strength is the adoption of standardised definitions and consistent diagnostic criteria based on carotid ultrasound findings, ensuring comparability across different regions and provinces. Benefiting from the large sample size and wide geographic coverage of our data, we were able to generate robust prevalence estimates at national, regional, and for the first time, provincial levels. Furthermore, we employed multilevel meta-regression models to account for heterogeneity across studies and an age- and sex- splitting method to enhance the data availability. Additionally, by integrating information on major associated factors such as smoking, hypertension, and diabetes, and distributing national case numbers according to the provincial distribution of these factors, we were able to provide a more nuanced understanding of the spatial heterogeneity in disease prevalence and burden, supporting more targeted public health planning.

Several limitations, however, should be acknowledged. First, although we used standardised diagnostic criteria and modelling methods to enhance comparability, heterogeneity in the original data sources, including differences in ultrasound equipment, operator expertise, and study populations, may have influenced prevalence estimates. Second, the national prevalence model included only age and sex as fixed effects, as other covariates (*e.g.* study year and economic region) were not found to be significant. While this model was statistically robust, a significant portion of between-study heterogeneity remained unexplained by these variables. This reinforces the need for our sub-national analyses to explore this variation more deeply. Third, while we incorporated major associated factors such as age, sex, smoking, hypertension, and diabetes into the distribution model for estimating regional and provincial case numbers, other important associated factors, including hyperlipidaemia, obesity, physical inactivity, and socioeconomic status, were not considered due to inconsistent definitions or insufficient data for a robust meta-analysis. The omission of these variables may have led to residual confounding and partially biased burden estimates at the subnational level. Fourth, our age-sex-splitting procedure relied on the national prevalence pattern, while the accuracy remains constrained by the uneven distribution of primary data across regions, particularly in less populous or remote areas, such as Xinjiang and Tibet, where data were limited. Fifth, we focused on estimating the overall prevalence of CAS and CP without distinguishing between asymptomatic and symptomatic cases or assessing the severity of carotid lesions – factors that are clinically important for risk stratification and management. Sixth, we were unable to provide prevalence estimates stratified by urban and rural settings due to data limitations in the included studies. Consequently, our provincial-level estimates may obscure intra-provincial disparities between urban and rural populations. Finally, the 95% CIs of our estimates did not fully capture the structural uncertainty arising from the imputation or modelling assumptions.

Our findings have important implications for public health policy and clinical practice in China. Atherosclerosis is a progressive, asymptomatic condition affecting multiple vascular territories, and the substantial prevalence of subclinical CAS and CP identified herein suggests a considerable future burden of cardiovascular morbidity and mortality [3,4,8–10,35]. Both CAS and CP, particularly CP, are strong predictors of subsequent vascular events, underscoring the urgent need for early detection, intensive risk factor modification, and proactive clinical management [8–10]. The provision of detailed prevalence estimates at national, regional, and provincial levels highlights marked geographical heterogeneity, reinforcing the necessity for region-specific prevention and control strategies rather than uniform national approaches. The observed variation in disease burden supports the prioritisation of resource allocation towards high-prevalence areas and the expansion of targeted screening programmes to facilitate earlier diagnosis and more effective risk stratification. The identification of modifiable associated factors, such as smoking, hypertension, and diabetes, further emphasises the critical role of comprehensive, integrated preventive strategies. Addressing persistent gaps in public awareness, access to preventive healthcare, and chronic disease management, particularly in rural and underserved populations, will be essential. Integration of CAS and CP surveillance and prevention into broader cardiovascular health frameworks, supported by primary care strengthening and community-based health education, may yield substantial reductions in the future burden of stroke and atherosclerotic vascular disease across China.

## CONCLUSIONS

This study robustly characterises the prevalence and distribution of CAS and CP in China, identifying considerable heterogeneity across regions and provinces in the process. The substantial burden of subclinical carotid disease signals an urgent public health challenge, with implications for future rates of cardiovascular morbidity and mortality. The identified associated factors, including smoking, hypertension, and diabetes, highlight critical targets for intervention. As the population continues to age and cardiovascular risk exposures persist, the prevalence of CAS and CP is likely to rise further. Strengthened efforts in early detection, risk factor management, and regionally adapted prevention strategies will be essential to reduce the long-term impact of atherosclerosis on the health of China’s population.

## Additional material


Online Supplementary Document
