# Supplementary material for: National, regional and provincial prevalence of carotid atherosclerosis and carotid plaque in Chinese adults: an updated systematic review and modelling analysis
Source: J Glob Health. 2026 Feb 27;16:04089. doi: 10.7189/jogh.16.04089 (PMC12945344; doi:10.7189/jogh.16.04089)
Supplement: Online Supplementary Document [file jogh-16-04089-s001.pdf]

Supplement to: Shan S, Wu J, Zhou J, Zhou L, Xu M, Zhu L, Rudan I, Song P. National, regional and provincial prevalence of carotid atherosclerosis and carotid plaque in Chinese adults: an updated systematic review and modelling analysis. J Glob Health. 2026;16:04089.

Content

Appendix 1. Search strategy to identify studies reporting the prevalence of carotid atherosclerosis and carotid plaque in China 3
Appendix 2. eMethods: The detailed description of stages used to derive national, regional, and provincial estimations of the prevalence of carotid atherosclerosis and carotid plaque ... 5
Appendix 3. Supplementary tables and figures 8
Table S1. The time-lag between study and publication in the included articles 8
Table S2. Quality assessment scale for rating the risk of bias 10
Table S3. Multilevel mixed-effects meta-regression models for prevalence patterns of the prevalence of carotid atherosclerosis and carotid plaque 11
Table S4. Age- and sex- adjusted meta-regression models of the prevalence of carotid atherosclerosis and carotid plaque 12
Table S5. Multilevel mixed-effects meta-regression models of the national prevalence of carotid atherosclerosis and carotid plaque 13
Table S6. Associated factors of carotid atherosclerosis and carotid plaque 14
Table S7. Four economic regions in the mainland of China 19
Table S8. Detailed characteristics of the included articles (n=82) 20
Table S9. Quality scores for assessing the risk of bias in the included articles (n=82) 25
Table S10. Main characteristics of the included articles (n=82) 27
Table S11. Estimated age- and sex-specific prevalence and case number of carotid atherosclerosis by economic regions in the mainland of China in 2020 28
Table S12. Estimated provincial prevalence and case number of carotid atherosclerosis in the mainland of China in 2020 30
Table S13. Estimated age- and sex-specific prevalence and case number of carotid plaque by economic regions in the mainland of China in 2020 32
Table S14. Estimated provincial prevalence and case number of carotid plaque in the mainland of China in 2020 34
Figure S1. Multilevel mixed-effects meta-regression models for prevalence patterns of the prevalence of carotid atherosclerosis and carotid plaque 36
Figure S2. Summary of associated factors of carotid atherosclerosis and carotid plaque 37
Appendix 4. Full list of the included articles (n=82) 38

Appendix 1. Search strategy to identify studies reporting the prevalence of carotid atherosclerosis and carotid plaque in China

Database	Access date	Subject category	Sub-database	Search terms	Publication date	Search method
**CNKI**	12/8/2024	Medicine & Public Health	Journal, Featured journal, Doctoral dissertation, Master dissertation	(TI%'颈动脉') AND (SU%'患病率'+'罹患率'+'现患率'+'流行'+'调查'+'现况')	16/4/2017-12/8/2024	Comprehensive search: subject, title, keywords and abstract
**Wanfang**	12/8/2024	Not applicable	Journal article, Dissertations	(题名:(颈动脉)) and (主题:(患病率) or 主题:(罹患率) or 主题:(现患率) or 主题:(流行) or 主题:(调查) or 主题:(现况))	2017-2024	Comprehensive search: subject (including title, keywords and abstract)
**VIP**	12/8/2024	Medicine & Public Health	All journals	M=(颈动脉)*M=(患病率+现患率+流行+调查+现况)	2017-2024	Comprehensive search: subject, title, keywords and abstract
**PubMed**	12/8/2024	Not applicable	Not applicable	((((carotid artery[Title/Abstract] OR carotid intima-media thickness[Title/Abstract] OR carotid thickening[Title/Abstract] OR carotid atherosclerosis[Title/Abstract] OR carotid atherosclerotic plaque[Title/Abstract] OR Carotid plaque[Title/Abstract] OR Carotid artery plaque[Title/Abstract] OR carotid arterial plaque[Title/Abstract] OR carotid Stenosis[Title/Abstract])) AND (China[Title/Abstract] OR Chinese[Title/Abstract])) AND (prevalen*[Title/Abstract] OR rate*[Title/Abstract] OR epidemiolog*[Title/Abstract])) AND ("2017/04/16"[Date - Publication] : "2024/08/12"[Date - Publication])	16/4/2017-12/8/2024	Comprehensive search: all fields
**Embase**	12/8/2024	Not applicable	Not applicable	#1 'carotid atherosclerosis'/exp<br>#2 'carotid atherosclerotic plaque'/exp OR 'carotid atherosclerotic plaque'<br>#3 ‘carotid artery disease’/exp OR ‘Carotid plaque’ OR ‘carotid artery obstruction’/exp<br>#4 'carotid artery plaque'/exp OR 'carotid artery plaque'<br>#5 ‘carotid arterial plaque’<br>#6 ‘carotid Stenosis’/exp OR ‘carotid Stenosis’<br>#7 'china'/exp<br>#8 'chinese'/exp<br>#9 'prevalence'/exp OR 'prevalen*'<br>#10 'rate*'<br>#11 ‘epidemiology’/exp OR ‘epidemiolog*’<br>#12 #1 OR #2 OR #3 OR #4 OR #5 OR #6<br>#13 #7 OR #8<br>#14 #9 OR #10 OR #11<br>#15 #12 AND #13 AND #14<br>#16 #15 AND [embase]/lim NOT ([embase]/lim AND [medline]/lim) AND [2017-2024]/py	01/01/2017-12/8/2024	Comprehensive search: all fields
**MEDLINE**	12/8/2024	Not applicable	Not applicable	#1 carotid atherosclerosis.mp. or exp Carotid Artery Diseases/<br>#2 carotid atherosclerotic plaque.mp.<br>#3 Carotid plaque.mp.<br>#4 Carotid artery plaque.mp.<br>#5 carotid arterial plaque.mp.<br>#6 exp Carotid Stenosis/	01/01/2017-12/8/2024	Comprehensive search: all fields

Database	Access date	Subject category	Sub-database	Search terms		Publication date	Search method
				#7	exp China/		
				#8	Chinese.mp.		
				#9	exp Prevalence/ or prevalen*.mp.		
				#10	rate*.mp.		
				#11	epidemiolog*.mp. or exp Epidemiology/		
				#12	1 or 2 or 3 or 4 or 5 or 6		
				#13	7 or 8		
				#14	9 or 10 or 11		
				#15	12 and 13 and 14		
				#16	limit 15 to yr="2017-Current"		

Notes: Number of records returned was 2,583. The access date was 12 August 2024.

Appendix 2. eMethods: The detailed description of stages used to derive national, regional, and provincial estimations of the prevalence of carotid atherosclerosis and carotid plaque

This section is a supplement to the Methods part in the main text.

Stage 1 Age-sex splitting for carotid atherosclerosis (CAS) and carotid plaque (CP)

Stage 1.1 Age- and sex-specific prevalence patterns for CAS and CP

We employed an age-sex-splitting method to enhance data availability for prevalence modelling. This procedure is based on the assumption of a shared, biologically-driven age- and sex- pattern of the disease and is used to split data reported in aggregate form. Community-based datapoints specifying age- and sex-specific prevalence for CAS and CP were extracted from the included articles. To provide a basis for age-sex- splitting for CAS and CP, we first adopted multilevel mixed-effects meta-regression models to explore the effects of age and sex on the prevalence of CAS and CP and to generate the corresponding "prevalence patterns". The following analysis was restricted to the 30-79-year age range, where sufficient data were available for model development. To enable the inclusion of zero cases as reported, zero cells were replaced with a value of 0.0005. The effect of datapoints clustering from the same study and the same province was controlled by adding study and province identification into the regression model as the random effect. Given that,

$$prevalence = p = \frac{CAS\ or\ CP\ cases}{number\ of\ participants}$$

Then the prevalence was stabilized by the logit link, and established as a function of average age and female proportion,

$$logit(p) = \ln\left(\frac{p}{1-p}\right) = \ln(odds) = \alpha + \beta_1 * Average\ age + \beta_2 * Female\ proportion + u_i$$

Therefore,

$$odds = \frac{p}{1-p} = e^{(\alpha+\beta_1*Average\ age+\beta_2*Female\ proportion+u_i)}$$

And,

$$prevalence = p = \frac{e^{(\alpha+\beta_1*Average\ age+\beta_2*Female\ proportion+u_i)}}{1+e^{(\alpha+\beta_1*Average\ age+\beta_2*Female\ proportion+u_i)}}$$

where α is the intercept term, β is the coefficient, and u_i is the random-effect.

Based on the above models, the "prevalence patterns" for CAS and CP were generated.

Stage 1.2 Age- and sex- splitting for CAS and CP

Based on the "prevalence patterns" for CAS and CP, we used an age-sex- splitting approach to convert data with standard age and sex groups. First, a sex split was performed on datapoints specified as "both" sex into male- and female-specific datapoints. The following equation was employed:

$$C_{A,s} = \left(\sum_{a\in A} R_{a,s} N_{a,s}\right) \cdot P_{A,s}/N_{A,s} \cdot \frac{C_{A,S}}{\sum_{s\in S}\left(\left(\sum_{a\in A} R_{a,s} N_{a,s}\right) \cdot P_{A,s}/N_{A,s}\right)}$$

In this equation, s is the specific sex (male or female), S is the set of sexes data is aggregated across, a is a one-year age group, A is the set of ages the data is aggregated across, $C_{A,S}$ is the reported total case number to be split, $R_{a,s}$ is the prevalence in age group a and sex s from "prevalence patterns", $N_{a,s}$ is the population in age group a and sex s based on the 2020 population census of China, $P_{A,s}$ is the proportion of sex s in study sample, $N_{A,s}$ is the population in age group A and sex s based on the 2020 population census of China, and $C_{A,s}$ is the split case number of CAS or CP in sex s.

Subsequently, datapoints with inconsistent age groups were split into uniform one-year age groups. Given the lack of precise age-specific distribution data in the included studies, we employed an exponential adjustment method with numerical optimization to simulate the age distributions align with both the national age distribution and the sample's average age.

We introduced an adjustment parameter adj to modify the national age distribution using an exponential function:

$$\boldsymbol{Adjusted\ P_a} = \frac{\boldsymbol{P_a \cdot e^{adj(a-\mu)}}}{\boldsymbol{\sum_{a \in A} P_a \cdot e^{adj(a-\mu)}}}$$

where $\boldsymbol{a}$ is a one-year age group, $\boldsymbol{A}$ is the set of ages the data is aggregated across, $\boldsymbol{adj}$ is the adjustment parameter, $\boldsymbol{\mu}$ is the average age of reported sample, $\boldsymbol{P_a}$ is the proportion of age $\boldsymbol{a}$ in the 2020 population census of China, and $\boldsymbol{Adjusted\ P_a}$ is the proportion of age $\boldsymbol{a}$ after adjustment. This adjustment increases the proportion of ages above $\boldsymbol{\mu}$ if $\boldsymbol{adj}$ >0 and increases the proportion of ages below $\boldsymbol{\mu}$ if $\boldsymbol{adj}$ <0.

The optimal value of $\boldsymbol{adj}$ were estimated using the Brent optimization method to minimize the squared difference between the average age of the adjusted distribution and the reported average age:

$$\boldsymbol{\min_{adj}\left(\sum_{a \in A} Adjusted\ P_a \cdot a - \mu\right)^2}$$

The optimization was constrained to $\boldsymbol{adj} \in [\boldsymbol{-10, 10}]$ to ensure numerical stability.

After obtaining the optimal $\boldsymbol{adj}$, we generated the adjusted age distribution $\boldsymbol{Adjusted\ P_a}$. We then sampled ages from this distribution to create a simulated population that aligns with both the national age distribution and the target mean age.

A sex split was then performed based on the adjusted age distribution:

$$\boldsymbol{C_a = R_a N_a \frac{C_A}{\sum_{a \in A} R_a N_a}}$$

In this equation, $\boldsymbol{a}$ is a one-year age group, $\boldsymbol{A}$ is the set of ages the data is aggregated across, $\boldsymbol{C_A}$ is the reported total case number in ages $\boldsymbol{A}$ to be split, $\boldsymbol{R_a}$ is the prevalence in age group $\boldsymbol{a}$ from "prevalence patterns", $\boldsymbol{N_a}$ is the population in age group $\boldsymbol{a}$ based on the simulated population, and $\boldsymbol{C_a}$ is the split case number of CAS or CP in age group $\boldsymbol{a}$.

After performing age-sex splitting, the case numbers for CAS and CP from various articles were divided into single-year age groups and separated by sex (male and female).

Stage 2: Epidemiological modelling of national, regional, and provincial prevalence and case number for CAS and CP

Stage 2.1 Age- and sex- specific prevalence of CAS and CP at national levels in 2020

To systematically address heterogeneity across studies and hierarchical data structures, multilevel mixed-effects meta-regression models were adopted, based on age-sex- split CAS and CP data. Datapoints falling outside the 95% prediction interval (PI) were classified as outliers and excluded from further analysis. We stabilized the prevalence by the logit link. Thus,

$$\boldsymbol{prevalence = p = \frac{e^{(\alpha+\beta_1 * x_1+\beta_2 * x_2 + \dots \beta_n * x_n + u_i)}}{1 + e^{(\alpha+\beta_1 * x_1+\beta_2 * x_2 + \dots \beta_n * x_n + u_i)}}}$$

where $\boldsymbol{\alpha}$ is the intercept term, $\boldsymbol{\beta}$ is the coefficient, $\boldsymbol{u_i}$ is the random-effect, and $\boldsymbol{x}$ is the variable.

The effects of study year and economic region on CAS and CP were first assessed independently, while neither of them was found to be significantly associated with the prevalence of CAS and CP. Thus, to estimate national prevalence of CAS and CP among people aged 30-79 years, we fitted multilevel multivariable mixed-effects meta-regression models with age and sex as fixed-effect variables, and study and province identification as the random-effects, respectively. Therefore,

$$\boldsymbol{prevalence = p = \frac{e^{(\alpha+\beta_1 * Age+\beta_2 * Sex + u_i)}}{1 + e^{(\alpha+\beta_1 * Age+\beta_2 * Sex + u_i)}}}$$

where $\boldsymbol{\alpha}$ is the intercept term, $\boldsymbol{\beta}$ is the coefficient, and $\boldsymbol{u_i}$ is the random-effect.

Based on the above models, the age-and sex-specific prevalence of CAS and CP was generated. The national case number of CAS and CP aged 30-79 years in 2020 were then generated by multiplying the estimated age- and sex-specific prevalence of CAS and CP in 2020 with the corresponding population data, obtained from the 2020 population census of China. This process established "national envelopes", representing the total number of CAS and CP cases aged 30-79 years across China in 2020 (without Taiwan, Hongkong, and Macau).

Stage 2.2 Meta-analysis of factors associated with CAS and CP

A subset of included articles additionally investigated associated factors of CAS and CP using multivariable analysis, and only factors that shared similar definitions were included. A random-effects (Restricted Maximum Likelihood) meta-analysis was conducted to synthesize the effects of associated factors with at least three informative datapoints.

Stage 2.3 Age- and sex- specific prevalence of CAS and CP at provincial levels in 2020

The national case number of CAS and CP aged 30-79 years in 2020 was then distributed into 31 provinces in the mainland of China through the "associated factor-based model", an "association-driven" approach initially proposed by Global Health Epidemiology Reference Group and has been widely used in estimating burden of disease at national, regional, and provincial levels. It uses the strength of association as well as distribution of key associated factors to allocate the national case "envelope" across regions and provinces. Three associated factors in ***Stage 2.2***, namely current smoking, hypertension, and diabetes, were selected incorporated into the "associated factor-based model". Then, the provincial numbers of CAS and CP cases ("province envelopes") were imputed by the following formula:

$$\boldsymbol{N_{province}} = \boldsymbol{POP_{province}} * \boldsymbol{Prev_{nation}} * \left(\boldsymbol{1} + \sum_{RF_1}^{RF_3} [(\boldsymbol{Prev_{RF_{province}}} - \boldsymbol{Prev_{RF_{nation}}}) * (\boldsymbol{OR_{RF}} - \boldsymbol{1})]\right)$$

Where $\boldsymbol{N_{province}}$ and $\boldsymbol{POP_{province}}$ are the number of CAS or CP cases and population size aged 30-79 years in each province or municipality. $\boldsymbol{Prev_{nation}}$ indicates the estimated national prevalence of CAS or CP generated in ***Stage 2.1***. $\boldsymbol{RF_1 - RF_3}$ are the three selected associated factors, namely current smoking, hypertension, and diabetes. $\boldsymbol{Prev_{RF_{province}}}$ and $\boldsymbol{Prev_{RF_{nation}}}$ are the prevalence of the three associated factors in each province or municipality and the mainland of China, which were obtained from previous large-scale studies. $\boldsymbol{OR_{RF}}$ is the synthesized OR of current smoking, hypertension, and diabetes from ***Stage 2.2***.

For each province, an "adjustment index" was used to ensure that the sum of provincial cases fit within "national envelopes". Then, the adjusted provincial prevalence of CAS or CP were calculated by the number of CAS or CP cases in each province or municipality divided by its corresponding population.

Stage 2.4 Age- and sex- specific prevalence of CAS or CP at regional levels in 2020

Finally, we developed "regional envelopes" for CAS or CP respectively, by summing the cases within each region (east China, central China, west China, and northeast China), and calculated prevalence of CAS or CP by the number of CAS or CP cases in each province or municipality divided by its corresponding population.

All analyses were conducted in R version 4.4.2 (https://www.r-project.org). *P*-values<0.05 were considered statistically significant.

Appendix 3. Supplementary tables and figures

Table S1. The time-lag between investigation and publication in the included articles

Article ID	Author	Publication year	Investigation year	Time-lag (years)
**A01**	Fan Jingwen, et al.	2024	2019	5
**A02**	Fu Jingzhu, et al.	2024	2019	5
**A03**	Wang Yuanping, et al.	2024	2020	4
**A04**	Yong Yufei, et al.	2024	2021	3
**A05**	Yu Jiayuan, et al.	2024	2021	3
**A06**	Zhang Xue.	2024	2022	2
**A07**	Zhou Naqi.	2024	2019	5
**A08**	Jiang Peng.	2023	2020	3
**A09**	Liu Chunxing, et al.	2023	2018	5
**A10**	Liu Dongjie.	2023	2020	3
**A11**	Pan Jia, et al.	2023	2021	2
**A12**	Tao Lijun, et al.	2023	2020	3
**A13**	Wang Shuwei, et al.	2023	2019	4
**A14**	Yang Tingting, et al.	2023	2018	5
**A15**	Yu Putian.	2023	2021	2
**A16**	Zeng Nimei, et al.	2023	2021	2
**A17**	Shen Qiuyu, et al.	2022	NA	NA
**A18**	Wang Anran.	2022	2019	3
**A19**	Zhu Lei.	2022	2021	1
**A20**	Dai Wen, et al.	2021	2019	2
**A21**	H. Shu-xia.	2021	2018	3
**A22**	He Zhili, et al.	2021	2019	2
**A23**	Huang Yuqing, et al.	2021	2018	3
**A24**	Liu Fang, et al.	2021	2019	2
**A25**	Lu Yu.	2021	2019	2
**A26**	Shen Zhiyuan, et al.	2021	2012	9
**A27**	Tang Qingwu, et al.	2021	2019	2
**A28**	Wu Tzuwei, et al.	2021	2015	6
**A29**	Xing Liying, et al.	2021	2018	3
**A30**	Yu Y, et al.	2021	2016	5
**A31**	Zhang Jie.	2021	2017	4
**A32**	Zhang Nan, et al.	2021	2018	3
**A33**	Guo Liping, et al.	2020	2019	1
**A34**	He Miao, et al.	2020	2015	5
**A35**	Huang Zhixin, et al.	2020	2019	1
**A36**	Liang Jun, et al.	2020	2017	3
**A37**	Ma Shouyuan, et al.	2020	2017	3
**A38**	Song Yang, et al.	2020	2019	1
**A39**	Yang Ying, et al.	2020	2012	8
**A40**	Yuan Qinghong.	2020	2019	1
**A41**	Fang Jianfei, et al.	2019	2017	2
**A42**	Lin Yanhua, et al.	2019	2016	3

Article ID	Author	Publication year	Investigation year	Time-lag (years)
**A43**	Wei Qiong'e, et al.	2019	NA	NA
**A44**	Xiao Meifang, et al.	2019	2017	2
**A45**	Zhou Pingan, et al.	2019	2012	7
**A46**	Chen Huan.	2018	2015	3
**A47**	Fan Xuesong, et al.	2018	2016	2
**A48**	Lin Chengguo, et al.	2018	2015	3
**A49**	Liu Qingxiang, et al.	2018	2015	3
**A50**	Shi Min.	2018	2014	4
**A51**	Tian Jing.	2018	2016	2
**A52**	Weng Genlong, et al.	2018	2014	4
**A53**	Xia Ronghui, et al.	2018	2016	2
**A54**	Yang Yingxia, et al.	2018	2015	3
**A55**	You Kai, et al.	2018	2016	2
**A56**	Zhang Benna, et al.	2018	2014	4
**A57**	Fan Fengjuan, et al.	2017	NA	NA
**A58**	Li Xiufeng, et al.	2017	2017	0
**A59**	Lu Jiqiang, et al.	2017	2015	2
**A60**	Robert Clarke, et al.	2017	2013	4
**A61**	Shang Jing, et al.	2017	2015	2
**A62**	Wang Xiujuan, et al.	2017	2017	0
**A63**	Zhang Lei, et al.	2017	2011	6
**A64**	Zhang Yong.	2017	2014	3
**A65**	Zhao Xiaoxia, et al.	2017	NA	NA
**A66**	Chen Xun, et.al.	2016	2014	2
**A67**	Xia Ting, et al.	2016	2012	4
**A68**	Zhang Yuma.	2016	2014	2
**A69**	Zhao Wei, et.al.	2016	2012	4
**A70**	Zhao Wei, et.al.	2016	2014	2
**A71**	Gao Yu, et.al.	2014	2014	0
**A72**	Guo Qiuxiang, et.al.	2014	NA	NA
**A73**	Lu Lu, et.al.	2014	NA	NA
**A74**	Ma Yifei.	2014	NA	NA
**A75**	Wang Li.	2014	2012	2
**A76**	Liu Xue.	2013	NA	NA
**A77**	Ma Xiangguo, et al.	2013	2012	1
**A78**	Qian Jiajia.	2013	2012	1
**A79**	Zhou Guirong, et al.	2013	2011	2
**A80**	Liu Beibei, et al.	2011	2010	1
**A81**	Sun Jing, et al.	2011	2010	1
**A82**	Yajuan Hu, et al.	2011	2010	1

Note: The average time-lag between investigation and publication was 2.90 years based on 74 articles with available data.

Table S2. Quality assessment scale for rating the risk of bias

Bias type	Low risk (score=2)	Moderate risk (score=1)	High risk (score=0)
Selection (sample population)	(1) Sample from general population, not a select group;<br>(2) Consecutive unselected population;<br>(3) Rationale for case and control selection explained.	(1) Sample selected from large population but selection criteria not defined;<br>(2) Sample selection ambiguous but may be representative;<br>(3) Rationale for cases and controls not explained;<br>(4) Eligibility criteria not explained;<br>(5) Analysis to adjust for sampling strategy bias.	(1) Highly select population making it difficult to generalise finding;<br>(2) Sample selection ambiguous and sample unlikely to be representative.
Selection (sample size)	(1) Sample size calculation performed and adequate.	(1) Sample size calculation performed and reasons for not meeting sample size given;<br>(2) Sample size calculation not performed but all eligible persons studied.	(1) Sample size estimation unclear or only sub-sample studied.
Selection (participation rate)	(1) High response rate (>85%).	(1) Moderate response rate (70-85%).	(1) Low response rate (<70%);<br>(2) Response rate not reported.
Performance bias (outcome assessment)	(1) Diagnosis using consistent criteria and direct examination.	(1) Assessment from administrative database or register;<br>(2) Assessment from hospital record or interviewer.	(1) Assessment from non-validated data or generic estimate from the overall population.
Performance bias (analytical methods to control for bias)	(1) Analysis appropriate for the type of sample (subgroup analysis/regression etc.).	(1) Analysis does not account for common adjustment.	(1) Data confusing.

Table S3. Multilevel mixed-effects meta-regression models for prevalence patterns of the prevalence of carotid atherosclerosis and carotid plaque

Variable	Number of data points	β (95% CI)	*P*-value	I^2
		**CAS**		
Intercept	53	-4.8707 (-5.3724, -4.4267)	**<0.0001**	
Average age	53	0.0851 (0.0814, 0.0889)	**<0.0001**	0.9974
Female proportion	53	-0.7694 (-0.8209, -0.7178)	**<0.0001**	
		**CP**		
Intercept	86	-5.1610 (-5.4373, -4.8847)	**<0.0001**	
Average age	86	0.0784 (0.0762, 0.0805)	**<0.0001**	0.9957
Female proportion	86	-0.4637 (-0.5108, -0.4166)	**<0.0001**	

Notes: CI, confidence interval; CAS, carotid atherosclerosis; CP, carotid plaque.

Table S4. Age- and sex- adjusted meta-regression models of the prevalence of carotid atherosclerosis and carotid plaque

Variable	Number of data points	β (95% CI)	*P*-value	I^2
		**CAS**		
Study year	1292	-0.0070 (-0.0955, 0.0815)	0.8769	0.9992
Study year category				
2010-2014	701	Reference		0.9991
2015-2019	582	0.0271 (-0.5888, 0.6431)	0.9312	
2020-2024	9	0.8959 (-0.4015, 2.1932)	0.1759	
Economic regions				
East	522	Reference		0.9986
Northeast	294	0.3556 (-0.8998, 1.6110)	0.5788	
Central	279	0.1496 (-1.0383, 1.3375)	0.8051	
West	197	-0.2686 (-1.6779, 1.1407)	0.7087	
		**CP**		
Study year	2033	-0.0179 (-0.0804, 0.0446)	0.5738	0.9984
Study year category				
2010-2014	1013	Reference		0.9985
2015-2019	809	0.1110 (-0.3228, 0.5447)	0.6161	
2020-2024	211	-0.1082 (-0.8128, 0.5963)	0.7634	
Economic regions				
East	1024	Reference		0.9986
Northeast	129	0.5099 (-0.4494, 1.4691)	0.2885	
Central	351	-0.2694 (-0.9148, 0.3760)	0.3848	
West	529	-0.2079 (-0.8078, 0.3921)	0.2374	

Notes: CI, confidence interval; CAS, carotid atherosclerosis; CP, carotid plaque.

Table S5. Multilevel mixed-effects meta-regression models of the national prevalence of carotid atherosclerosis and carotid plaque

Variable		Number of data points	β (95% CI)	*P*-value	I^2
**CAS**					
Intercept		1292	-5.1815 (-5.5584, -4.8046)	**<0.0001**	
Age		1292	0.0814 (0.0812, 0.0815)	**<0.0001**	
Sex					0.9994
	Female	690	Reference		
	Male	602	0.7540 (0.7516, 0.7563)	**<0.0001**	
**CP**					
Intercept		2033	-5.2271 (-5.4272, -5.0269)	**<0.0001**	
Age		2033	0.0779 (0.0778, 0.0780)	**<0.0001**	
Sex					0.9984
	Female	1033	Reference		
	Male	1000	0.4704 (0.4683, 0.4724)	**<0.0001**	

Notes: CI, confidence interval; CAS, carotid atherosclerosis; CP, carotid plaque.

Table S6. Associated factors of carotid atherosclerosis and carotid plaque

Article ID	Author	Publication year	Sample size
**CAS**			
**Age (per 1 year)**			
A05	Yu Jiayuan, et al.	2024	419
A07	Zhou Naqi.	2024	1631
A10	Liu Dongjie.	2023	5041
A14	Yang Tingting, et al.	2023	2578
A33	Guo Liping, et al.	2020	2188
A68	Zhang Yuma.	2016	2291
A75	Wang Li.	2014	1253

Forest Plot

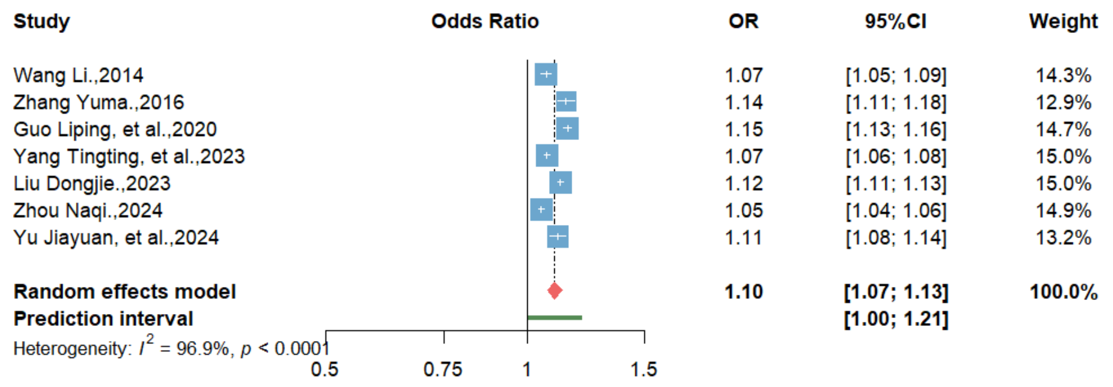

Funnel Plot

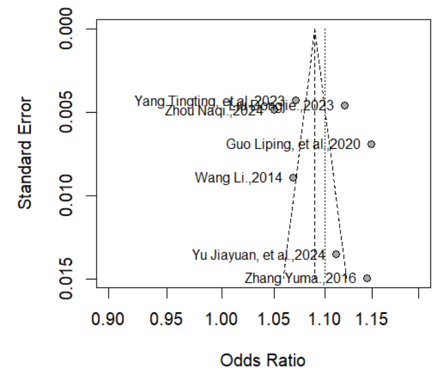

Sensitivity analysis

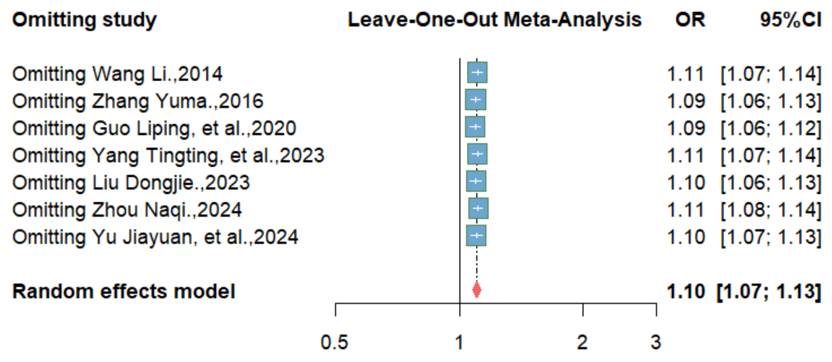

Article ID	Author	Publication year	Sample size
**Sex (Male)**			
A02	Fu Jingzhu, et al.	2024	10733975
A07	Zhou Naqi.	2024	1631
A10	Liu Dongjie.	2023	5041
A14	Yang Tingting, et al.	2023	2578
A18	Wang Anran.	2022	8811
A29	Xing Liying, et al.	2021	5838
A33	Guo Liping, et al.	2020	2188
A35	Huang Zhixin, et al.	2020	1560
A54	Yang Yingxia, et al.	2018	2197
A68	Zhang Yuma.	2016	1253
A75	Wang Li.	2014	2291

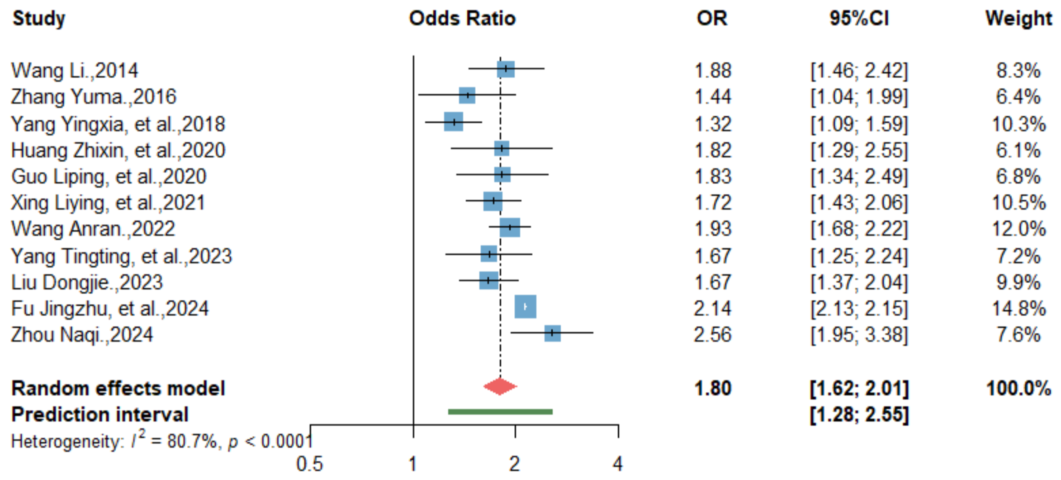

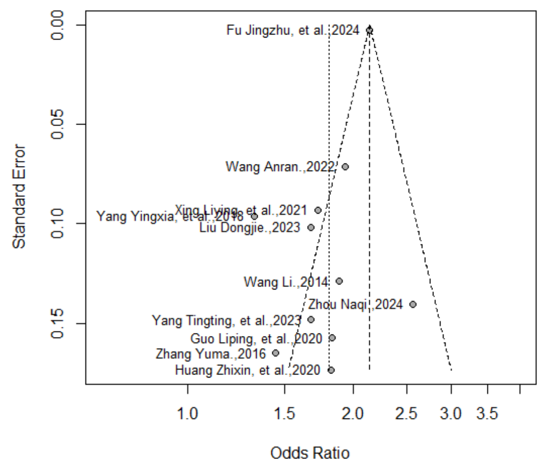

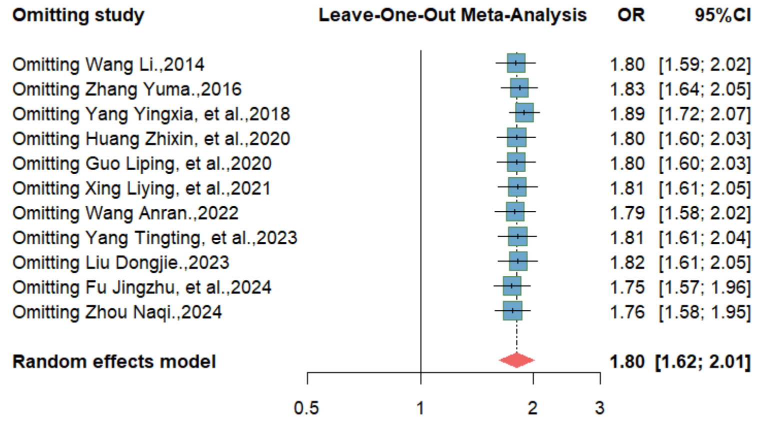

Article ID	Author	Publication year	Sample size
**Current smoking**			
A18	Wang Anran.	2022	8811
A54	Yang Yingxia, et al.	2018	2197
A57	Fan Fengjuan, et al.	2017	280
A75	Wang Li.	2014	2291

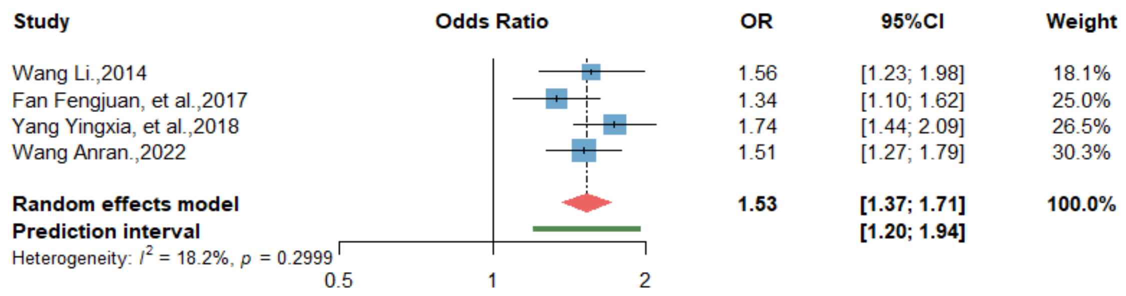

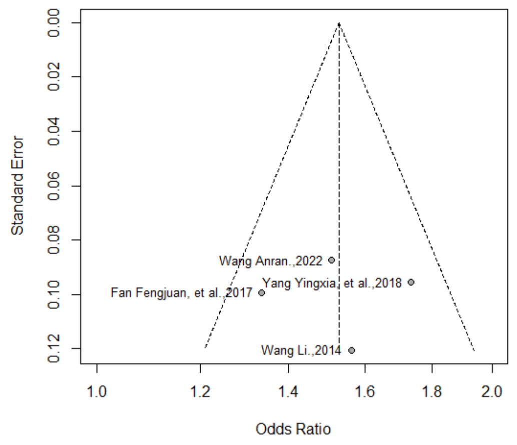

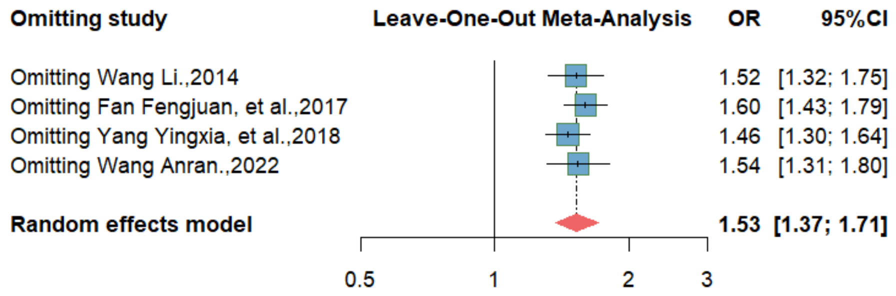

Article ID	Author	Publication year	Sample size
**Current drinking**			
A18	Wang Anran.	2022	8811
A29	Xing Liying, et al.	2021	5838
A75	Wang Li.	2014	2291

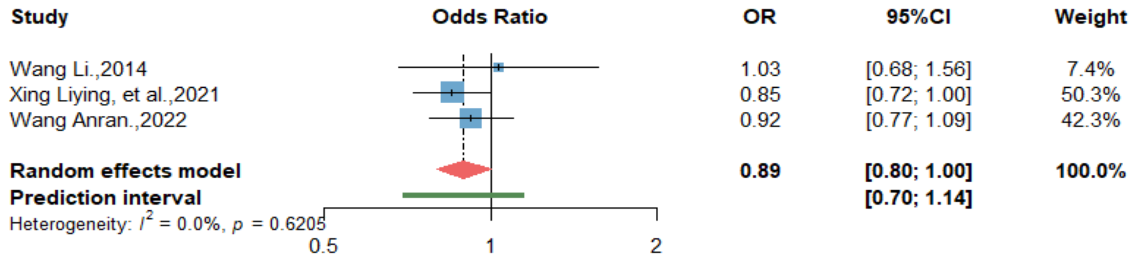

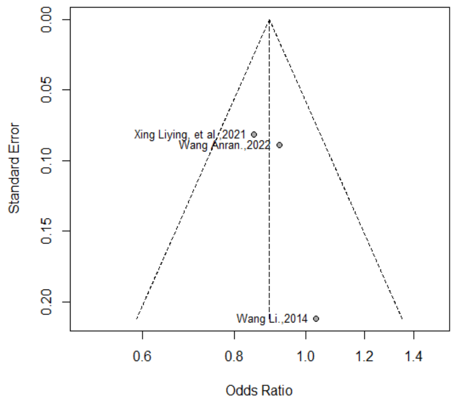

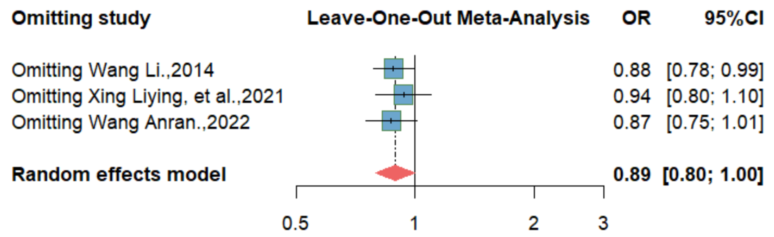

Article ID	Author	Publication year	Sample size
**Hypertension**			
A02	Fu Jingzhu, et al.	2024	10733975
A05	Yu Jiayuan, et al.	2024	419
A07	Zhou Naqi.	2024	1631
A10	Liu Dongjie.	2023	5041
A14	Yang Tingting, et al.	2023	2578
A15	Yu Putian.	2023	220

Article ID	Author	Publication year	Sample size
A18	Wang Anran.	2022	8811
A29	Xing Liying, et al.	2021	5838
A54	Yang Yingxia, et al.	2018	2197
A57	Fan Fengjuan, et al.	2017	280
A68	Zhang Yuma.	2016	1253
A75	Wang Li.	2014	2291

Forest Plot

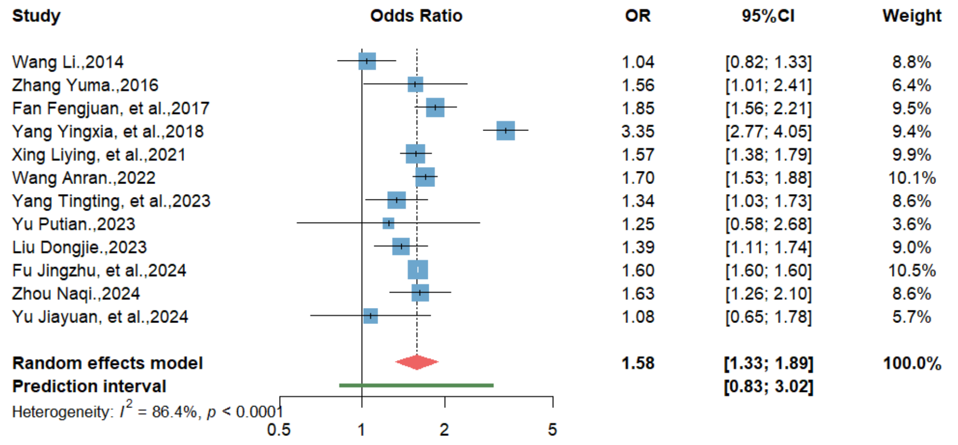

Funnel Plot

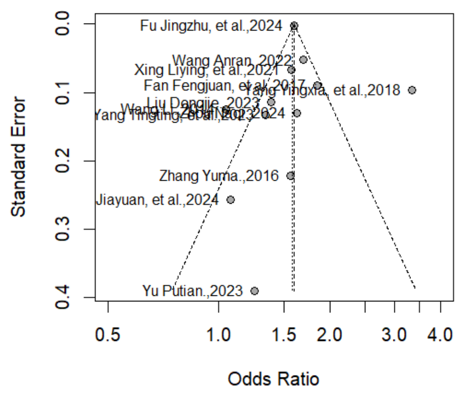

Sensitivity analysis

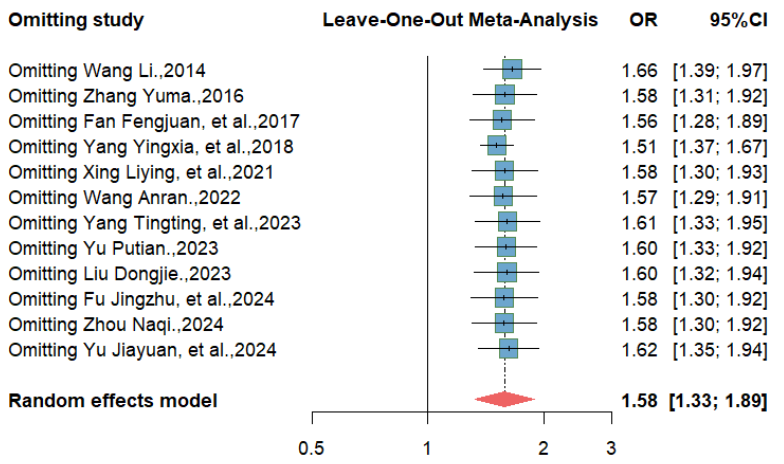

Diabetes

Article ID	Author	Publication year	Sample size
A02	Fu Jingzhu, et al.	2024	10733975
A08	Jiang Peng.	2023	983
A18	Wang Anran.	2022	8811
A54	Yang Yingxia, et al.	2018	2197
A75	Wang Li.	2014	2291

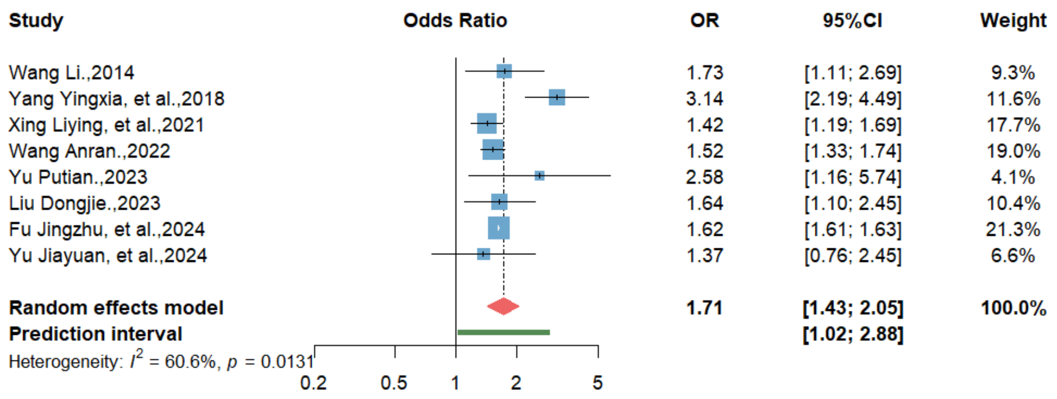

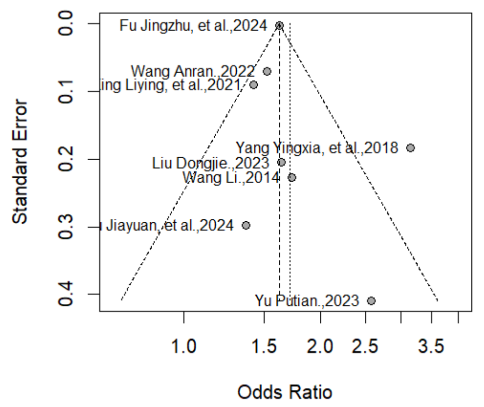

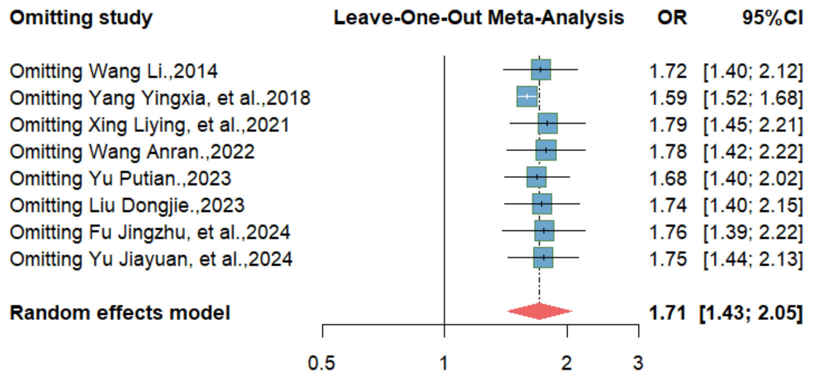

Dyslipidemia

Article ID	Author	Publication year	Sample size
A02	Fu Jingzhu, et al.	2024	10733975
A15	Yu Putian.	2023	220
A18	Wang Anran.	2022	8811
A57	Fan Fengjuan, et al.	2017	280
A75	Wang Li.	2014	2291

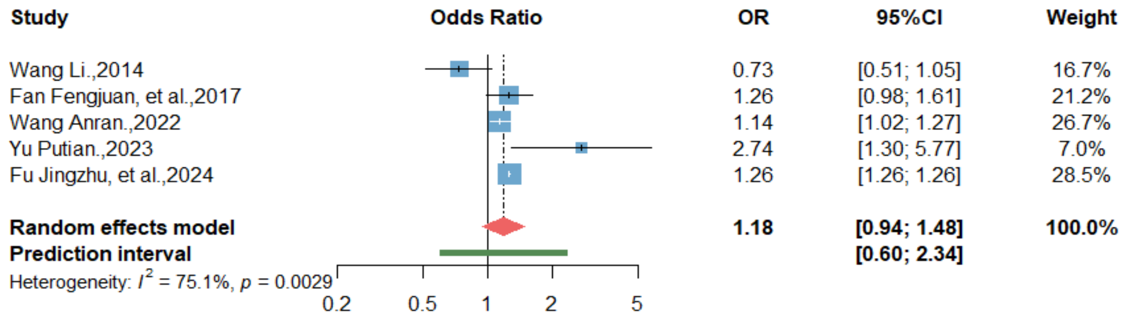

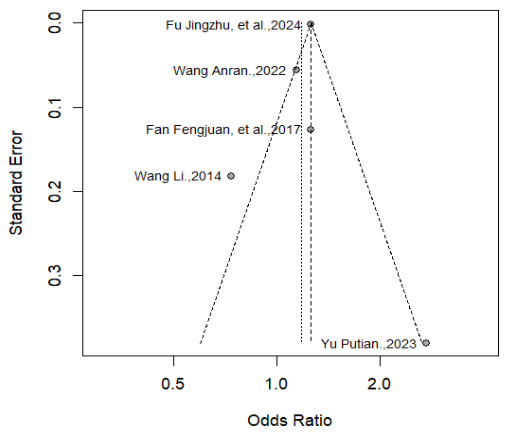

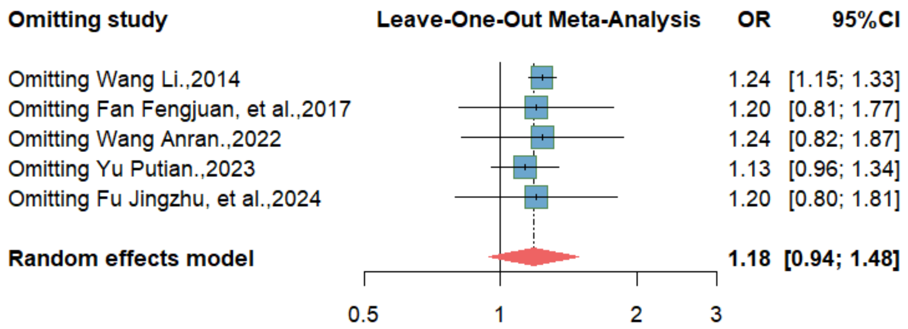

HDL-c (per 1 mmol/L)

Article ID	Author	Publication year	Sample size
A07	Zhou Naqi.	2024	1631
A14	Yang Tingting, et al.	2023	2578
A33	Guo Liping, et al.	2020	2188

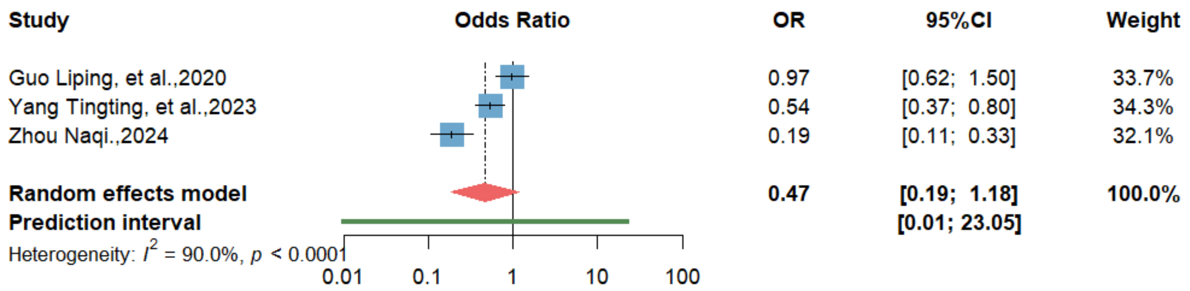

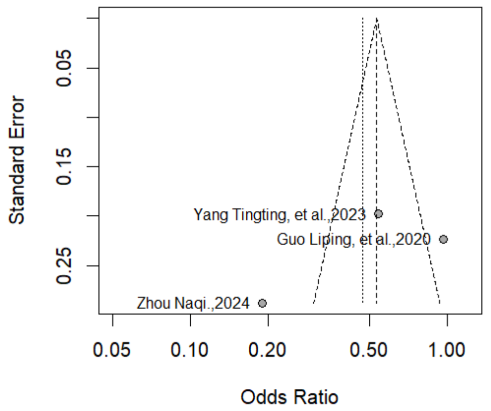

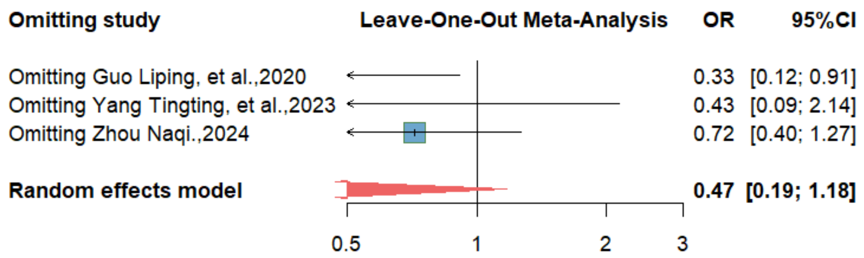

UA (per 1 μmol/L)

Article ID	Author	Publication year	Sample size
A02	Fu Jingzhu, et al.	2024	10733975
A07	Zhou Naqi.	2024	1631
A10	Liu Dongjie.	2023	5041
A33	Guo Liping, et al.	2020	2188

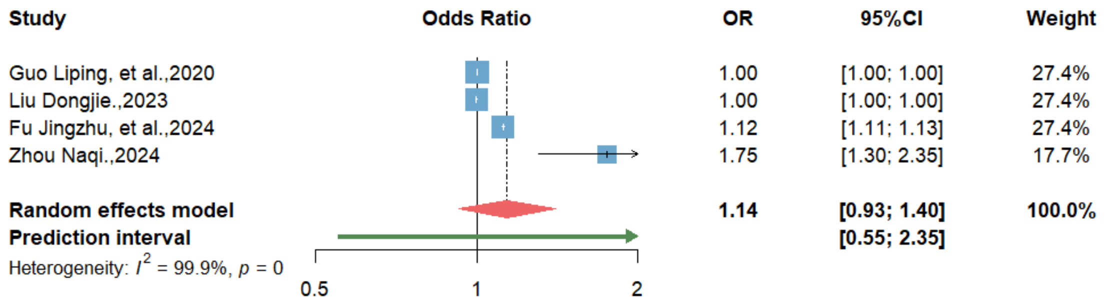

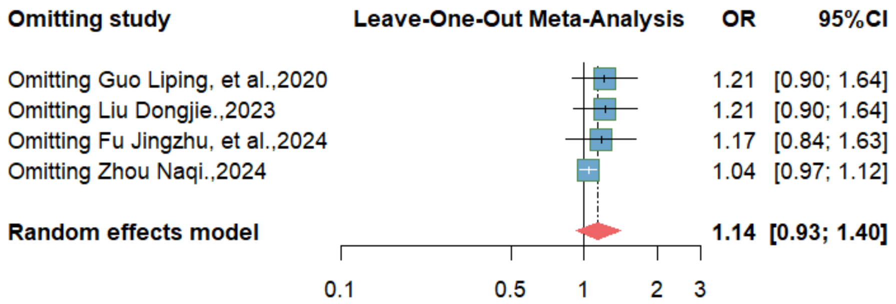

Article ID	Author	Publication year	Sample size	Forest Plot	Funnel Plot	Sensitivity analysis

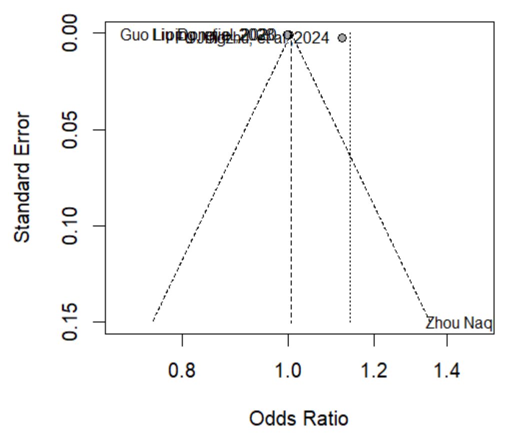

Hcy (>15μmmol/L)

Article ID	Author	Publication year	Sample size
A15	Yu Putian.	2023	220
A18	Wang Anran.	2022	8811
A75	Wang Li.	2014	2291

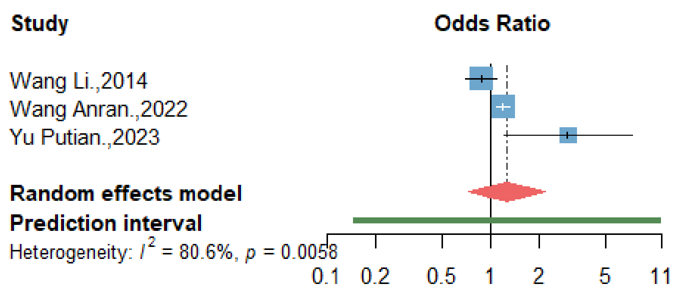

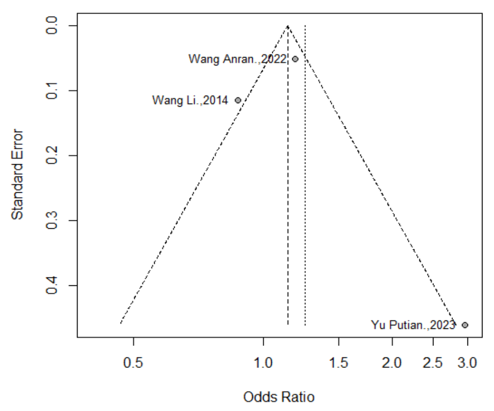

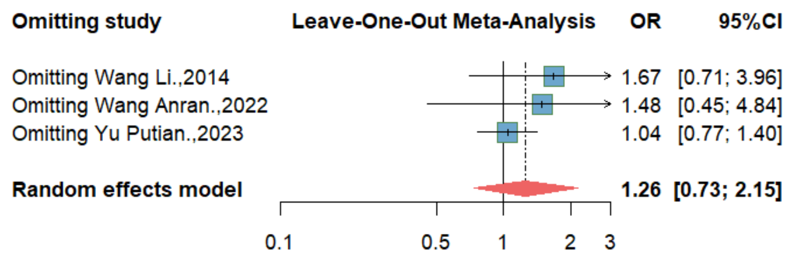

CP

Age (per 1 year)

Article ID	Author	Publication year	Sample size
A08	Jiang Peng.	2023	983
A10	Liu Dongjie.	2023	5041
A25	Lu Yu.	2021	1861
A28	Wu Tzuwei, et al.	2021	3908
A36	Liang Jun, et al.	2020	1381
A59	Lu Jiqiang, et al.	2017	1014

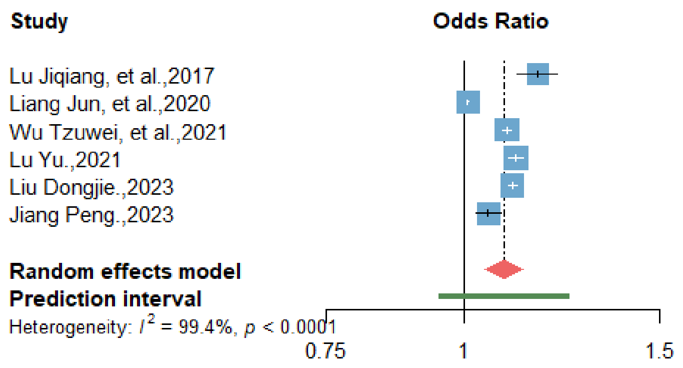

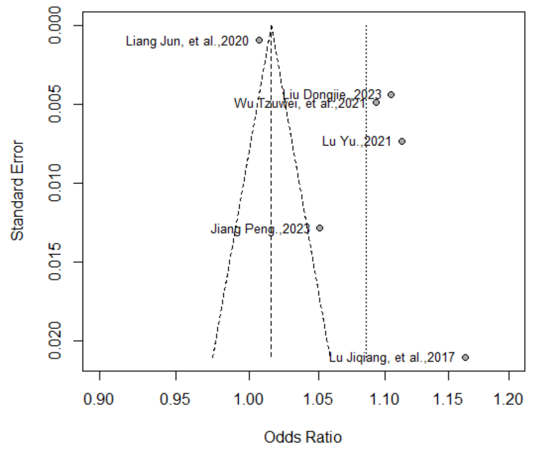

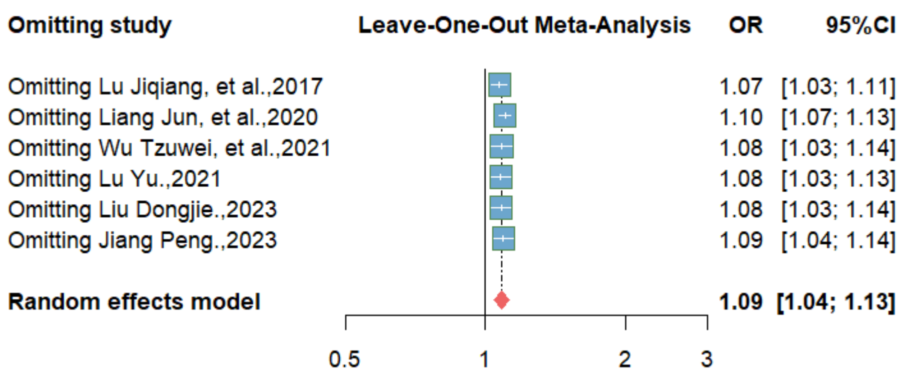

Sex (Male)

Article ID	Author	Publication year	Sample size
A02	Fu Jingzhu, et al.	2024	10733975
A10	Liu Dongjie.	2023	5041
A18	Wang Anran.	2022	8811
A25	Lu Yu.	2021	1861
A28	Wu Tzuwei, et al.	2021	3908
A36	Liang Jun, et al.	2020	1381
A41	Fang Jianfei, et al.	2019	2394
A59	Lu Jiqiang, et al.	2017	1014

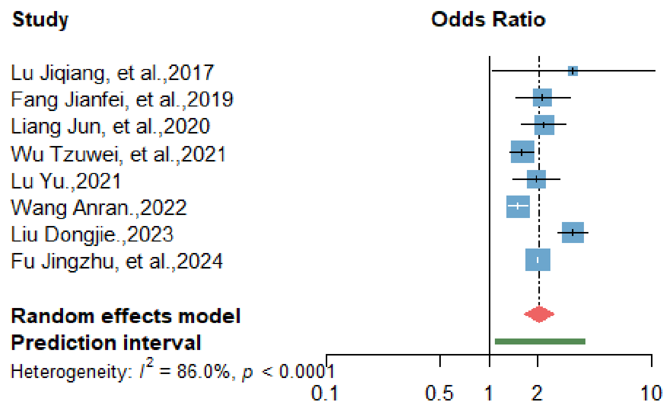

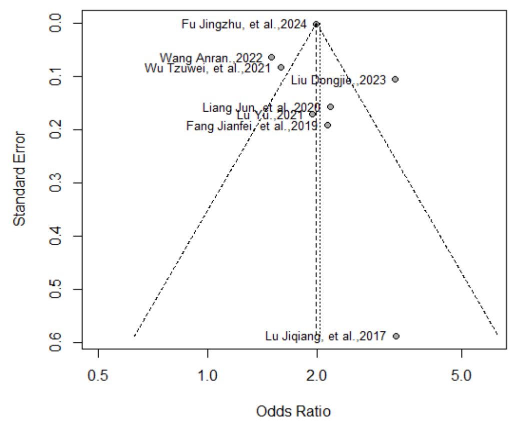

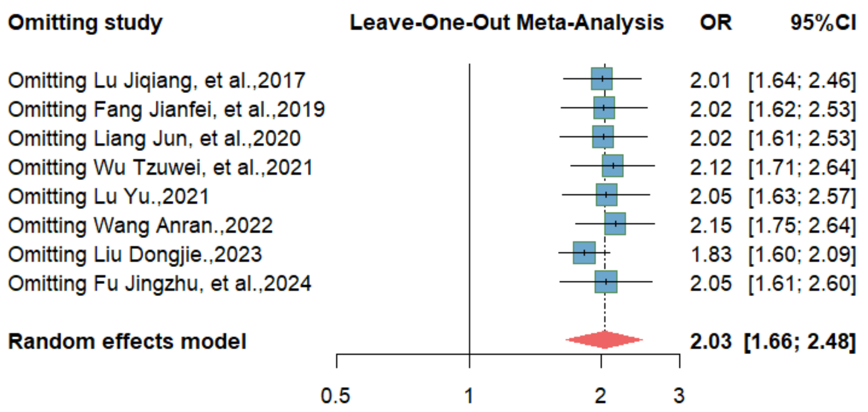

Current smoking

Article ID	Author	Publication year	Sample size
A18	Wang Anran.	2022	8811
A28	Wu Tzuwei, et al.	2021	3908

Article ID	Author	Publication year	Sample size	Forest Plot	Funnel Plot	Sensitivity analysis
A31	Zhang Jie.	2021	2021	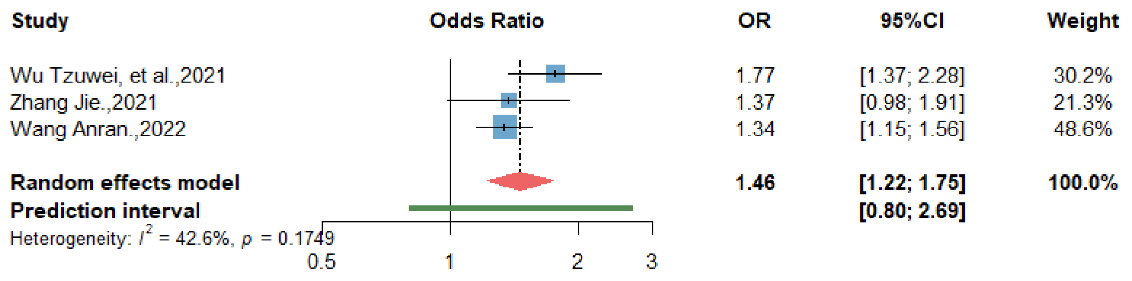	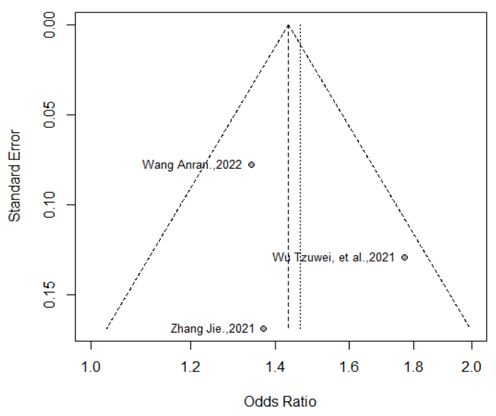	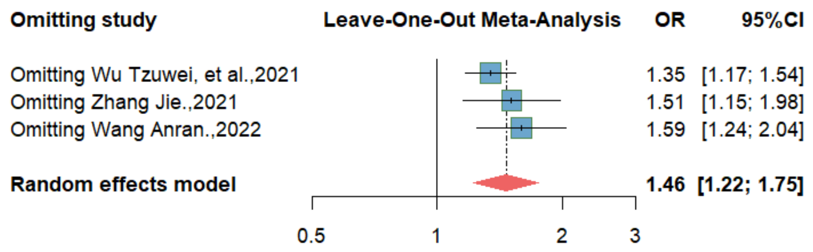
**Current drinking**						
A18	Wang Anran.	2022	8811	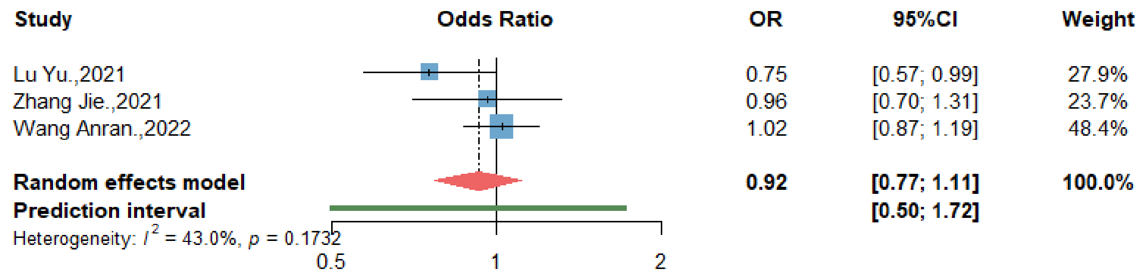	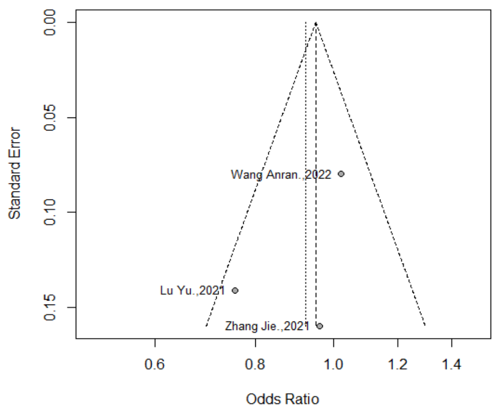	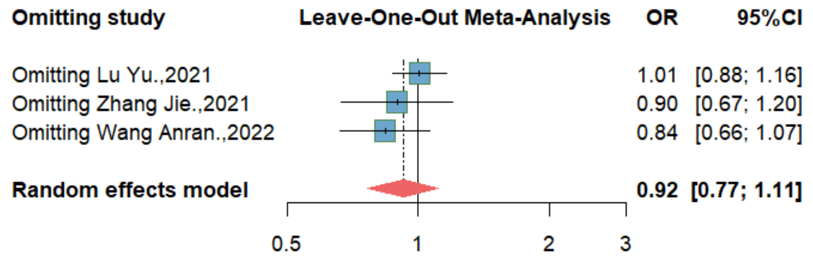
A25	Lu Yu.	2021	1861			
A31	Zhang Jie.	2021	2021			
**Hypertension**						
A02	Fu Jingzhu, et al.	2024	10733975	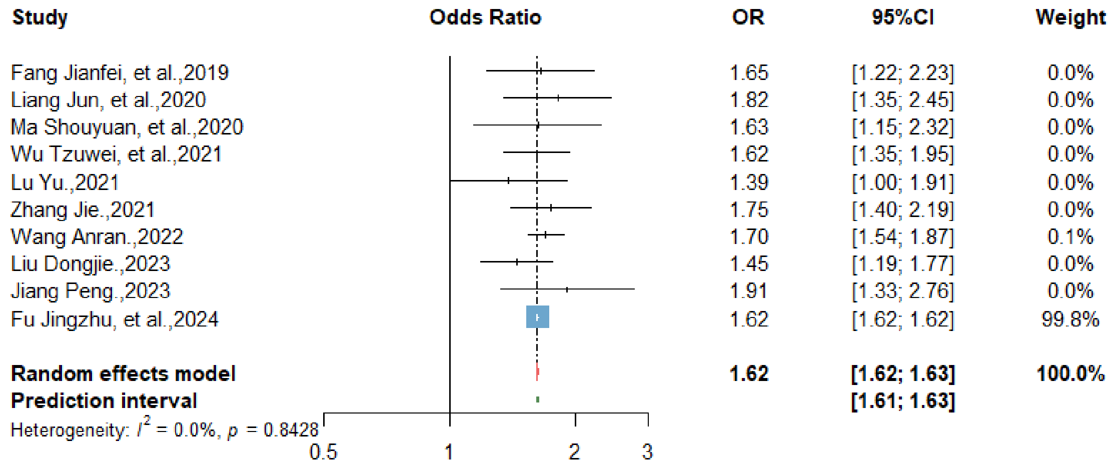	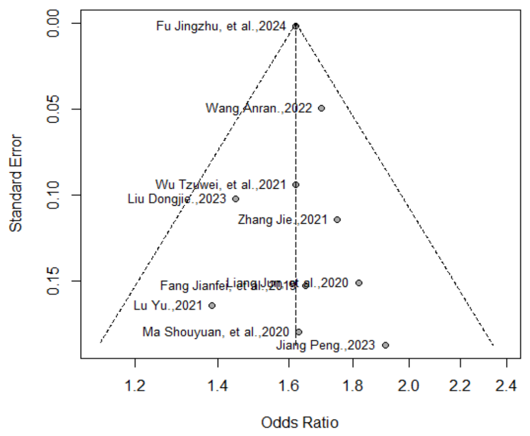	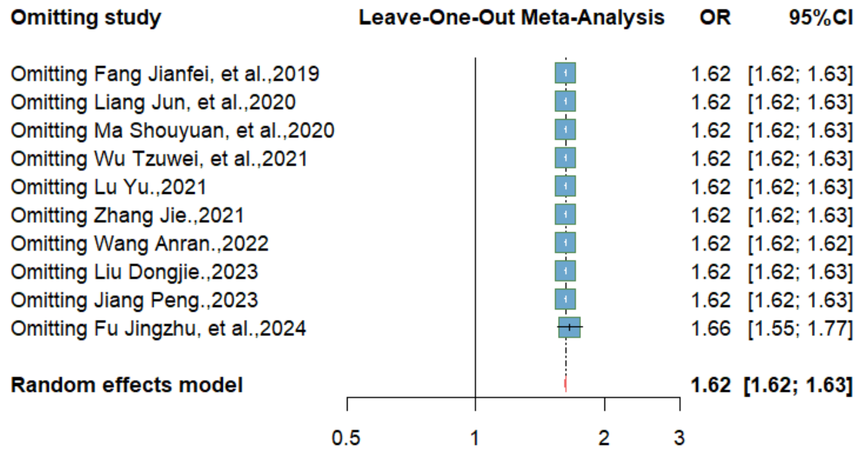
A08	Jiang Peng.	2023	983			
A10	Liu Dongjie.	2023	5041			
A18	Wang Anran.	2022	8811			
A25	Lu Yu.	2021	1861			
A28	Wu Tzuwei, et al.	2021	3908			
A31	Zhang Jie.	2021	2021			
A36	Liang Jun, et al.	2020	1381			
A37	Ma Shouyuan, et al.	2020	716			
A41	Fang Jianfei, et al.	2019	2394			
**Diabetes**						
A02	Fu Jingzhu, et al.	2024	10733975	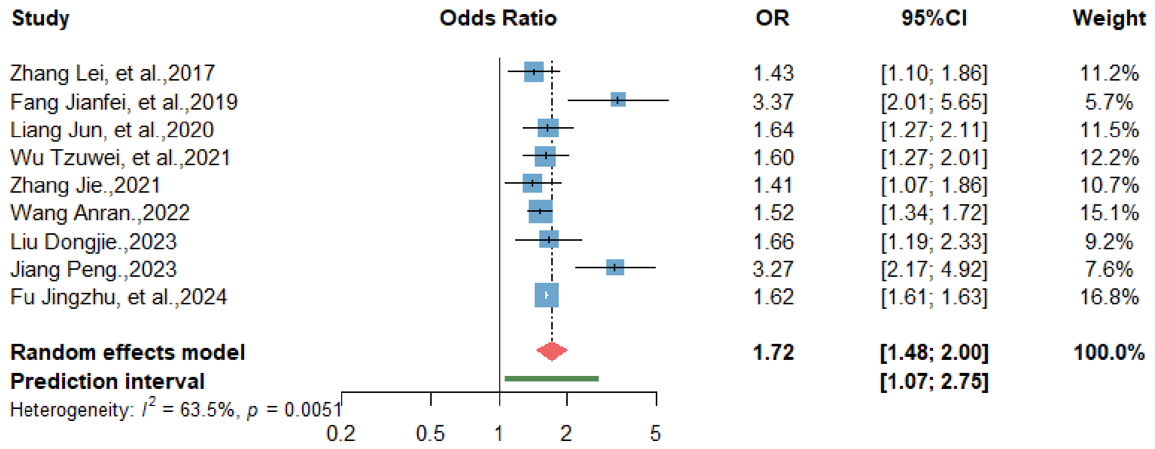	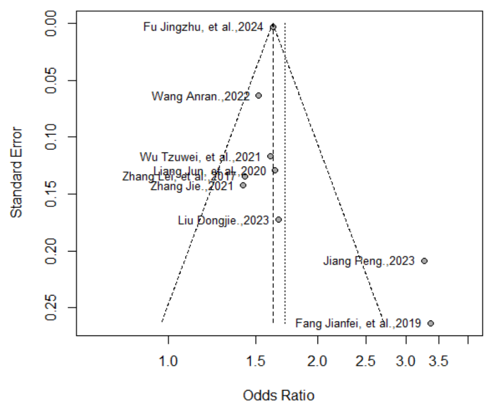	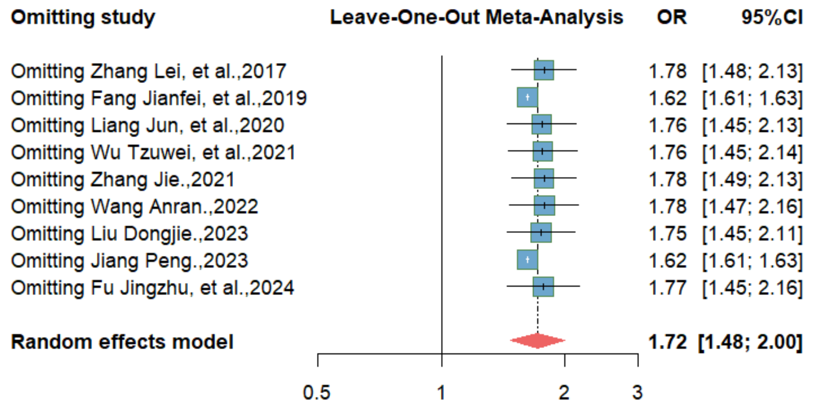
A08	Jiang Peng.	2023	983			
A10	Liu Dongjie.	2023	5041			
A18	Wang Anran.	2022	8811			
A28	Wu Tzuwei, et al.	2021	3908			
A31	Zhang Jie.	2021	2021			
A36	Liang Jun, et al.	2020	1381			
A41	Fang Jianfei, et al.	2019	2394			
A63	Zhang Lei, et al.	2017	5994			
**Dyslipidemia**						
A02	Fu Jingzhu, et al.	2024	10733975	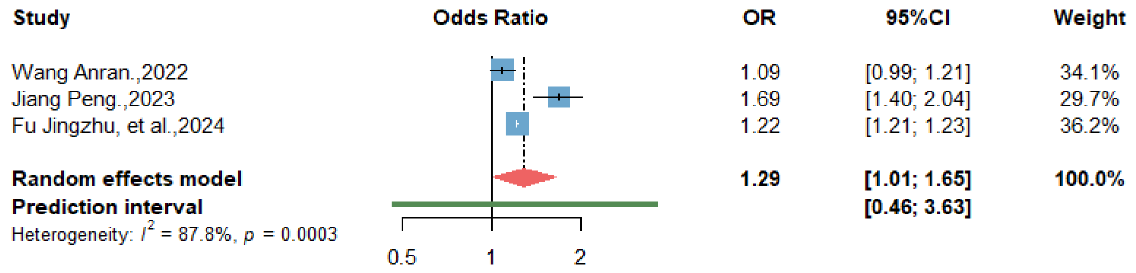	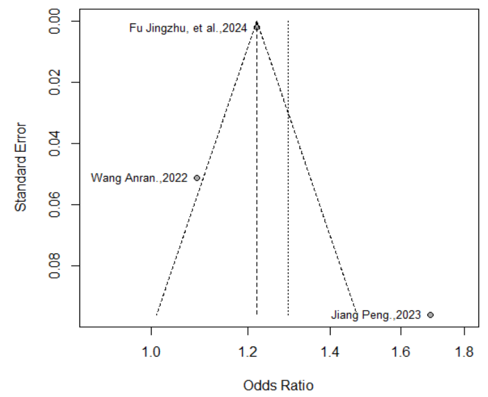	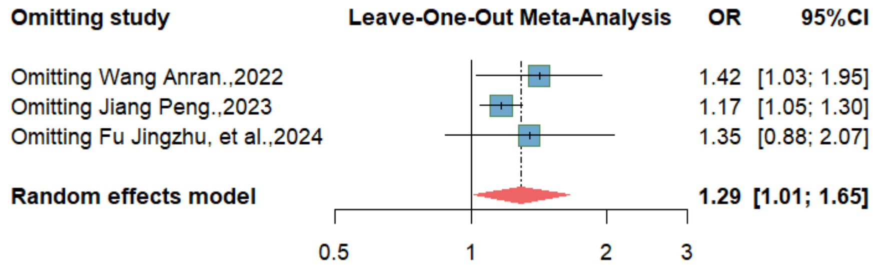
A08	Jiang Peng.	2023	983			
A18	Wang Anran.	2022	8811			

Article ID	Author	Publication year	Sample size	Forest Plot	Funnel Plot	Sensitivity analysis
				**Fatty liver**		
A19	Liu Dongjie.	2023	5041			
A25	Lu Yu.	2021	1861			
A41	Fang Jianfei, et al.	2019	2394	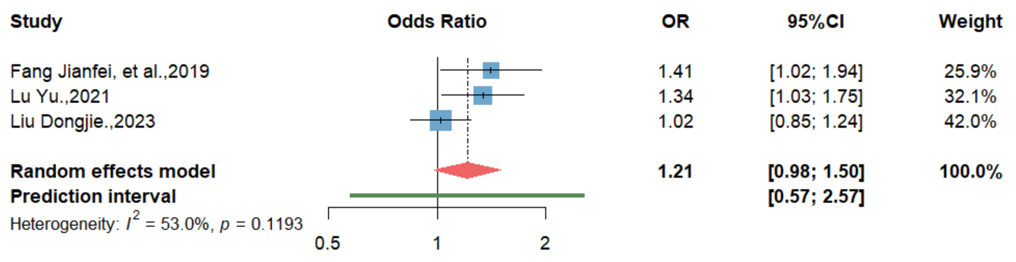	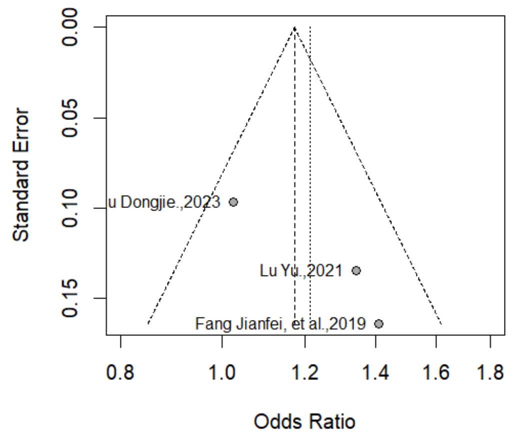	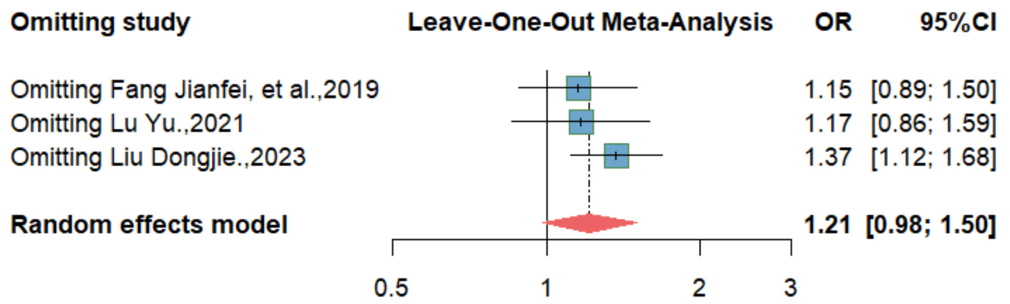

Notes: CAS, carotid atherosclerosis; CP, carotid plaque; HDL-c, high-density lipoprotein cholesterol; UA, uric acid; Hcy, homocysteine; OR, odds ratio; CI, confidence interval.

Table S7. Four economic regions in the mainland of China

Economic region	Included provinces
East China	Beijing Municipality, Tianjin Municipality, Hebei province, Shanghai Municipality, Jiangsu province, Zhejiang province, Fujian province, Shandong province, Guangdong province, Hainan province
Central China	Shanxi province, Anhui province, Jiangxi province, Henan province, Hubei province, Hunan province
West China	Inner Mongolia Autonomous Region, Guangxi Zhuang Autonomous Region, Chongqing Municipality, Sichuan province, Guizhou province, Yunnan province, Tibet Autonomous Region, Shaanxi province, Gansu province, Qinghai province, Ningxia Hui Autonomous Region, Xinjiang Uyghur Autonomous Region
Northeast China	Liaoning province, Jilin province, Heilongjiang province

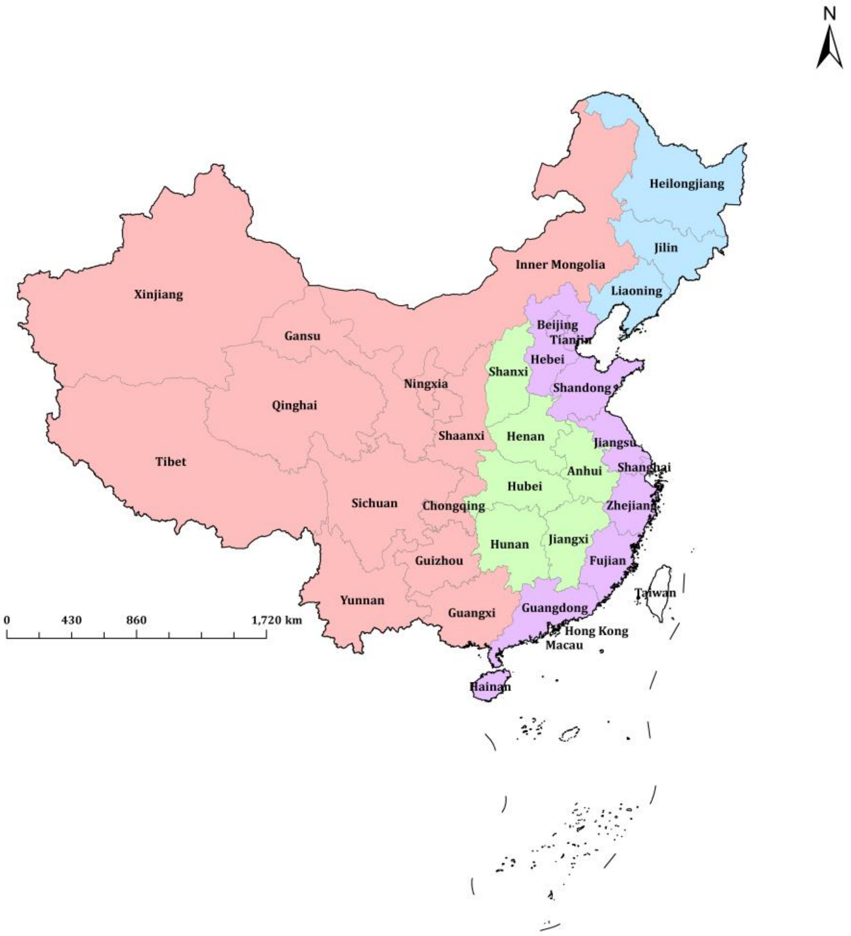

Table S8. Detailed characteristics of the included articles (n=82)

Article ID	Author	Publication year	Province	Setting	Investigation date	Community-based or health check-based	Age range	Female proportion	Sample size	NO. of CAS	NO. of CP
A01	Fan Jingwen, et al.	2024	Henan	Mixed	2017-2020	Health check-based	NA	33.97%	17396		10742
A02	Fu Jingzhu, et al.	2024	31 Province (without Tibet)	Mixed	2017.1.1-2022.6.30	Health check-based	20+	45.39%	10733975	3394208	2254135
A03	Wang Yuanping, et al.	2024	Fujian	Mixed	2019.1-2021.6	Community-based	35-74	64.51%	7263	1648	904
A04	Yong Yufei, et al.	2024	Shandong	Mixed	2019.1-2022.12	Health check-based	45-60	52.4%	1105		328
A05	Yu Jiayuan, et al.	2024	Jilin	Mixed	2020.9-2022.12	Health check-based	24-81	41.77%	419	253	
A06	Zhang Xue.	2024	Beijing	Urban	2022.1-2022.12	Community-based	65+	72.0%	515	498	388
A07	Zhou Naqi.	2024	Inner Mongolia	Mixed	2017.1-2021.12	Health check-based	25-75	28.94%	1631	884	
A08	Jiang Peng.	2023	Sichuan	Rural	2020.8-2020.9	Community-based	50+	57.07%	983		171
A09	Liu Chunxing, et al.	2023	Jiangsu	Mixed	2018	Health check-based	18-92	37.37%	20836		1581
A10	Liu Dongjie.	2023	Liaoning	Mixed	2019.1-2021.12	Health check-based	NA	39.63%	5041	2609	1655
A11	Pan Jia, et al.	2023	China	Mixed	2021.1-2021.12	Community-based	18+	1.01%	3169		511
A12	Tao Lijun, et al.	2023	Guangxi	Mixed	2019.11-2021.11	Community-based	30-74	60.29%	481		135
A13	Wang Shuwei, et al.	2023	Hunan	Mixed	2018.6-2020.12	Health check-based	18-70	28.54%	5211		1164
A14	Yang Tingting, et al.	2023	Henan	Mixed	2018.1-2018.7	Health check-based	18+	30.06%	2578	707	
A15	Yu Putian.	2023	Liaoning	Urban	2021.11-2022.1	Health check-based	60-85	55.91%	220	176	146
A16	Zeng Nimei, et al.	2023	Jiangsu	Mixed	2021	Health check-based	NA	32.59%	12317	4991	3038
A17	Shen Qiuyu, et al.	2022	Shanghai	Mixed	NA	Health check-based	40+	0.00%	1080	143	126

Article ID	Author	Publication year	Province	Setting	Investigation date	Community-based or health check-based	Age range	Female proportion	Sample size	NO. of CAS	NO. of CP
A18	Wang Anran.	2022	Henan	Rural	2018.1-2019.12	Community-based	40+	61.20%	8811	5420	4572
A19	Zhu Lei.	2022	Shandong	Mixed	2021.2-2021.10	Health check-based	21+	46.00%	300		33
A20	Dai Wen, et al.	2021	Henan	Mixed	2019.1-2019.11	Health check-based	21-99	10.79%	2809		609
A21	H. Shu-xia.	2021	Guangxi	Mixed	2018	Health check-based	70-92	43.53%	170	147	141
A22	He Zhili, et al.	2021	Jiangxi	Mixed	2019.1-2019.12	Community-based	40+	54.85%	25622		5652
A23	Huang Yuqing, et al.	2021	Guangdong	Mixed	2017.1-2018.12	Community-based	54-66	63.31%	2652		1383
A24	Liu Fang, et al.	2021	Henan	Mixed	2019.4-2020.4	Health check-based	14+	35.39%	729	485	
A25	Lu Yu.	2021	Guangdong	Mixed	2019.1-2019.6	Health check-based	25-85	29.50%	1861		427
A26	Shen Zhiyuan, et al.	2021	Hebei	Rural	2012.6-2012.7	Community-based	30-94	58.28%	2598		728
A27	Tang Qingwu, et al.	2021	Jiangxi	Mixed	2018.8-2020.6	Community-based	40+	51.67%	360	203	102
A28	Wu Tzuwei, et al.	2021	Taiwan	Urban	2010.9-2020.5	Community-based	40-74	64.82%	3908		1344
A29	Xing Liying, et al.	2021	Liaoning	Rural	2017.9-2018.5	Community-based	40+	60.67%	5838		2333
A30	Yu Y, et al.	2021	Jilin	Mixed	2016.1-2016.3	Community-based	40+	59.87%	3688	1911	1794
A31	Zhang Jie.	2021	Shandong	Rural	2017.1	Community-based	40+	52.25%	2021		774
A32	Zhang Nan, et al.	2021	Liaoning	Mixed	2018.1-2018.6	Health check-based	18+	42.23%	2740	1350	
A33	Guo Liping, et al.	2020	Beijing	Mixed	2019.1-2019.12	Health check-based	24-96	29.6%	2188	1380	
A34	He Miao, et al.	2020	31 Province (without Tibet)	Mixed	2014.10-2015.11	Community-based	40+	53.62%	84880	41964	31803
A35	Huang Zhixin, et al.	2020	Guangdong	Mixed	2018-2019	Community-based	35-91	66.60%	1560	876	
A36	Liang Jun, et al.	2020	Shanghai	Urban	2017	Health check-based	35-87	55.3%	1381		755
A37	Ma Shouyuan, et al.	2020	Beijing	Mixed	2017	Health check-based	18+	55.17%	716		289

Article ID	Author	Publication year	Province	Setting	Investigation date	Community-based or health check-based	Age range	Female proportion	Sample size	NO. of CAS	NO. of CP
A38	Song Yang, et al.	2020	Beijing	Mixed	2019.2-2019.5	Health check-based	30-86	41.00%	300	182	117
A39	Yang Ying, et al.	2020	Beijing	Mixed	2011.12-2012.4	Community-based	40+	64.19%	1078		336
A40	Yuan Qinghong.	2020	Hunan	Mixed	2018.5-2019.4	Health check-based	34-68	NA	800		84
A41	Fang Jianfei, et al.	2019	Zhejiang	Mixed	2016.7-2017.8	Health check-based	18-94	32.41%	2394	527	284
A42	Lin Yanhua, et al.	2019	Henan	Mixed	2015.1-2017.12	Health check-based	18+	31.18%	3214		1421
A43	Wei Qiong'e, et al.	2019	Yunnan	Mixed	NA	Health check-based	18+	NA	173	19	13
A44	Xiao Meifang, et al.	2019	Hunan	Mixed	2017.1-2017.12	Health check-based	32-88	40.41%	6238	1595	
A45	Zhou Pingan, et al.	2019	Hebei	Mixed	2010-2014	Community-based	40+	44.70%	8933		3582
A46	Chen Huan.	2018	Hunan	Rural	2015.11-2016.1	Community-based	40-81	52.77%	866	379	253
A47	Fan Xuesong, et al.	2018	Beijing	Mixed	2015.11-2016.2	Health check-based	30-86	46.55%	174	92	60
A48	Lin Chengguo, et al.	2018	Zhejiang	Urban	2015	Community-based	35-65	68.6%	1607		118
A49	Liu Qingxiang, et al.	2018	Beijing	Mixed	2015	Community-based	40-98	66.42%	4387	654	
A50	Shi Min.	2018	Tianjin	Rural	2014.4-2014.6	Community-based	45+	58.06%	3946		1489
A51	Tian Jing.	2018	Chongqing	Mixed	2015.7-2016.5	Health check-based	45-87	38.90%	545	308	210
A52	Weng Genlong, et al.	2018	Jiangsu	Mixed	2013.9-2014.7	Community-based	60-80	54.73%	3126	1183	981
A53	Xia Ronghui, et al.	2018	Beijing	Mixed	2016.1-2016.6	Health check-based	55-96	53.72%	914		637
A54	Yang Yingxia, et al.	2018	Gansu	Mixed	2014.8-2017.1	Health check-based	45-89	43.79%	2197	990	
A55	You Kai, et al.	2018	Beijing	Mixed	2015.12-2016.6	Community-based	50-80	62.5%	2445	1419	1287
A56	Zhang Benna, et al.	2018	Beijing	Urban	2014.5-2014.7	Community-based	40+	63.92%	3830		2171
A57	Fan Fengjuan, et al.	2017	Guangdong	Mixed	NA	Community-based	60-82	43.57%	280	151	116

Article ID	Author	Publication year	Province	Setting	Investigation date	Community-based or health check-based	Age range	Female proportion	Sample size	NO. of CAS	NO. of CP
A58	Li Xiufeng, et al.	2017	Beijing	Mixed	2016-2017	Health check-based	22-95	42.33%	1578	471	
A59	Lu Jiqiang, et al.	2017	Jiangsu	Mixed	2015.1-2015.10	Health check-based	23-72	23.67%	1014		98
A60	Robert Clarke, et al.	2017	Hunan, Zhejiang, Sichuan, Jiangsu, Shandong, Gansu, Henan, Guangxi, Hainan, Heilongjiang	Mixed	2013-2014	Community-based	40-89	61.74%	24822		7695
A61	Shang Jing, et al.	2017	Shandong	Mixed	2014.9-2015.7	Community-based	45+	48.7%	10182	7618	5688
A62	Wang Xiujuan, et al.	2017	Guangdong	Mixed	2016.9-2017.9	Health check-based	30-70	39.48%	613		63
A63	Zhang Lei, et al.	2017	Hebei	Mixed	2010.4-2011.10	Health check-based	40-88	44.21%	5994		2597
A64	Zhang Yong.	2017	Hebei	Mixed	2013.7-2014.8	Health check-based	40-82	51.94%	3398	180	
A65	Zhao Xiaoxia, et al.	2017	Gansu	Mixed	NA	Health check-based	46+	47.6%	511	355	236
A66	Chen Xun, et.al.	2016	Guangxi	Urban	2013.1-2015.3	Community-based	40-77	45.45%	5789	1646	
A67	Xia Ting, et al.	2016	Gansu	Mixed	2011.6-2013.12	Community-based	45+	54.07%	1770		459
A68	Zhang Yuma.	2016	Inner Mongolia	Mixed	2014.4-2014.11	Community-based	40+	61.53%	1253	577	372
A69	Zhao Wei, et.al.	2016	Beijing	Urban	2012.1-2012.12	Community-based	55-85	57.98%	752		223
A70	Zhao Wei, et.al.	2016	Tianjin	Rural	2014.4-2015.1	Community-based	45+	58.83%	3789		1574
A71	Gao Yu, et.al.	2014	Beijing	Mixed	2014.1-2014.5	Community-based	45+	52.08%	1227	697	554
A72	Guo Qiuxiang, et.al.	2014	Inner Mongolia and Beijing	Mixed	NA	Community-based	55-75	42.38%	4084	3663	
A73	Lu Lu, et.al.	2014	Liaoning	Mixed	NA	Community-based	40+	75.16%	9300	5510	
A74	Ma Yifei.	2014	Zhejiang	Rural	NA	Health check-based	60-81	42.93%	559	302	233
A75	Wang Li.	2014	Shanxi	Rural	2012.8-2012.11	Community-based	55+	55.65%	2291	1747	1114
A76	Liu Xue.	2013	Liaoning	Urban	NA	Community-based	40+	75.81%	8449	4882	

Article ID	Author	Publication year	Province	Setting	Investigation date	Community-based or health check-based	Age range	Female proportion	Sample size	NO. of CAS	NO. of CP
A77	Ma Xiangguo, et al.	2013	Heilongjiang	Rural	2011.10-2013.6	Community-based	30-80	NA	400	136	
A78	Qian Jiajia.	2013	Jiangsu	Urban	2011.10-2012.11	Community-based	20-59	51.74%	201		54
A79	Zhou Guirong, et al.	2013	Beijing	Rural	2010.5-2012.10	Community-based	47-94	61.91%	2216	1933	1288
A80	Liu Beibei, et al.	2011	Beijing	Mixed	2010.7-2010.9	Community-based	55+	54.66%	1557	1376	1024
A81	Sun Jing, et al.	2011	Beijing	Urban	2009.9-2010.6	Community-based	60+	59.69%	2121	1375	1179
A82	Yajuan Hu, et al.	2011	Beijing	Urban	2010	Community-based	29-41	43.96%	1126		38

Notes: NA, not available; CAS, carotid atherosclerosis; CP, carotid plaque.

Table S9. Quality scores for assessing the risk of bias in the included articles (n=82)

Article ID	Author	Publication year	Sample population	Sample size	Participation rate	Outcome assessment	Analytical methods	Total scores
**A01**	Fan Jingwen, et al.	2024	2	0	0	1	2	5
**A02**	Fu Jingzhu, et al.	2024	2	1	0	1	2	6
**A03**	Wang Yuanping, et al.	2024	2	0	0	1	2	5
**A04**	Yong Yufei, et al.	2024	2	0	0	1	1	4
**A05**	Yu Jiayuan, et al.	2024	2	0	0	2	2	6
**A06**	Zhang Xue.	2024	2	0	0	2	1	5
**A07**	Zhou Naqi.	2024	2	0	0	2	2	6
**A08**	Jiang Peng.	2023	2	0	0	2	2	6
**A09**	Liu Chunxing, et al.	2023	2	0	0	1	2	5
**A10**	Liu Dongjie.	2023	2	0	0	2	2	6
**A11**	Pan Jia, et al.	2023	1	1	0	2	2	6
**A12**	Tao Lijun, et al.	2023	2	0	0	2	2	6
**A13**	Wang Shuwei, et al.	2023	2	0	0	1	2	5
**A14**	Yang Tingting, et al.	2023	2	0	0	1	1	4
**A15**	Yu Putian.	2023	1	0	0	2	1	4
**A16**	Zeng Nimei, et al.	2023	2	0	0	2	2	6
**A17**	Shen Qiuyu, et al.	2022	1	0	0	2	1	4
**A18**	Wang Anran.	2022	2	0	2	2	2	8
**A19**	Zhu Lei.	2022	2	0	0	2	1	5
**A20**	Dai Wen, et al.	2021	1	0	0	2	2	5
**A21**	H. Shu-xia.	2021	1	0	0	2	1	4
**A22**	He Zhili, et al.	2021	2	0	0	2	1	5
**A23**	Huang Yuqing, et al.	2021	2	1	0	1	2	6
**A24**	Liu Fang, et al.	2021	2	0	0	1	2	5
**A25**	Lu Yu.	2021	2	0	0	2	2	6
**A26**	Shen Zhiyuan, et al.	2021	2	1	0	2	1	6
**A27**	Tang Qingwu, et al.	2021	2	0	0	2	1	5
**A28**	Wu Tzuwei, et al.	2021	2	0	0	2	2	6
**A29**	Xing Liying, et al.	2021	2	1	0	2	2	7
**A30**	Yu Y, et al.	2021	2	1	2	2	2	9
**A31**	Zhang Jie.	2021	2	0	0	2	2	6
**A32**	Zhang Nan, et al.	2021	2	0	0	2	2	6
**A33**	Guo Liping, et al.	2020	1	1	2	2	2	8
**A34**	He Miao, et al.	2020	2	1	0	2	2	7
**A35**	Huang Zhixin, et al.	2020	2	0	0	2	2	6
**A36**	Liang Jun, et al.	2020	2	0	0	1	1	4
**A37**	Ma Shouyuan, et al.	2020	2	0	0	2	2	6
**A38**	Song Yang, et al.	2020	2	0	0	2	2	6
**A39**	Yang Ying, et al.	2020	2	1	0	2	2	7
**A40**	Yuan Qinghong.	2020	2	0	0	2	1	5
**A41**	Fang Jianfei, et al.	2019	2	0	0	2	2	6
**A42**	Lin Yanhua, et al.	2019	2	0	0	1	2	5
**A43**	Wei Qiong'e, et al.	2019	1	0	0	2	1	4
**A44**	Xiao Meifang, et al.	2019	2	0	0	2	2	6

Article ID	Author	Publication year	Sample population	Sample size	Participation rate	Outcome assessment	Analytical methods	Total scores
**A45**	Zhou Pingan, et al.	2019	2	1	0	2	2	7
**A46**	Chen Huan.	2018	2	0	0	2	2	6
**A47**	Fan Xuesong, et al.	2018	2	0	0	2	2	6
**A48**	Lin Chengguo, et al.	2018	2	1	0	2	2	7
**A49**	Liu Qingxiang, et al.	2018	2	0	0	2	2	6
**A50**	Shi Min.	2018	2	0	0	2	2	6
**A51**	Tian Jing.	2018	2	0	0	2	2	6
**A52**	Weng Genlong, et al.	2018	2	0	0	2	1	5
**A53**	Xia Ronghui, et al.	2018	1	0	0	2	2	5
**A54**	Yang Yingxia, et al.	2018	1	0	0	1	2	4
**A55**	You Kai, et al.	2018	2	1	2	1	1	7
**A56**	Zhang Benna, et al.	2018	2	0	0	2	2	6
**A57**	Fan Fengjuan, et al.	2017	2	0	0	2	2	6
**A58**	Li Xiufeng, et al.	2017	1	0	0	1	2	4
**A59**	Lu Jiqiang, et al.	2017	1	0	0	2	2	5
**A60**	Robert Clarke, et al.	2017	2	1	1	1	1	6
**A61**	Shang Jing, et al.	2017	2	1	2	2	1	8
**A62**	Wang Xiujuan, et al.	2017	2	0	0	2	1	5
**A63**	Zhang Lei, et al.	2017	1	0	0	2	2	5
**A64**	Zhang Yong.	2017	2	0	0	2	2	6
**A65**	Zhao Xiaoxia, et al.	2017	1	1	0	2	1	5
**A66**	Chen Xun, et.al.	2016	2	1	0	2	2	7
**A67**	Xia Ting, et al.	2016	1	1	0	2	2	6
**A68**	Zhang Yuma.	2016	1	1	0	2	2	6
**A69**	Zhao Wei, et.al.	2016	1	1	0	2	1	5
**A70**	Zhao Wei, et.al.	2016	1	1	1	2	2	7
**A71**	Gao Yu, et.al.	2014	1	1	0	2	1	5
**A72**	Guo Qiuxiang, et.al.	2014	1	1	0	2	1	5
**A73**	Lu Lu, et.al.	2014	1	1	0	2	2	6
**A74**	Ma Yifei.	2014	1	1	0	2	1	5
**A75**	Wang Li.	2014	1	1	0	2	2	6
**A76**	Liu Xue.	2013	1	0	1	2	2	6
**A77**	Ma Xiangguo, et al.	2013	1	1	0	2	1	5
**A78**	Qian Jiajia.	2013	1	0	0	2	2	5
**A79**	Zhou Guirong, et al.	2013	1	1	0	2	1	5
**A80**	Liu Beibei, et al.	2011	1	0	0	2	2	5
**A81**	Sun Jing, et al.	2011	1	0	0	2	1	4
**A82**	Yajuan Hu, et al.	2011	2	0	0	2	2	6

Table S10. Summary of the included articles (n=82)

Characteristics	Articles reported CAS or CP prevalence (n=82)
	N (%)
**CAS**	47 (57.32)
**CP**	65 (79.27)
**Publication year**	
2010-2019	42 (51.22)
2019-2024	40 (48.78)
**Economic region***	
East	44 (53.66)
Central	12 (14.63)
West	11 (13.41)
Northeast	9 (10.98)
**Community-based or health check-based**	
Community-based	44 (53.66)
Health check-based	38 (46.34)
**Quality score**	
9	1 (1.22)
8	3 (3.66)
7	8 (9.76)
6	34 (41.46)
<=5	36 (43.90)

Notes: CAS, carotid atherosclerosis; CP, carotid plaque. *Five articles provided prevalence data based on multiple provinces.

Table S11. Estimated age- and sex-specific prevalence and case number of carotid atherosclerosis by economic regions in the mainland of China in 2020

Age group	Prevalence (%, 95% CI)			Case number (million, 95% CI)		
	Both	Male	Female	Both	Male	Female
			East			
**30-39 year**	12.72 (9.14, 17.40)	16.52 (11.97, 22.33)	8.58 (6.06, 12.02)	12.63 (9.08, 17.27)	8.54 (6.19, 11.55)	4.08 (2.88, 5.72)
**40-49 year**	24.93 (18.73, 32.32)	31.53 (24.07, 40.07)	17.97 (13.09, 24.16)	20.42 (15.34, 26.48)	13.25 (10.12, 16.84)	7.17 (5.22, 9.64)
**50-59 year**	40.72 (32.36, 49.63)	49.34 (40.17, 58.55)	31.90 (24.38, 40.50)	34.88 (27.72, 42.52)	21.37 (17.39, 25.35)	13.52 (10.33, 17.16)
**60-69 year**	60.36 (51.48, 68.64)	68.81 (60.38, 76.14)	52.04 (42.72, 61.26)	35.42 (30.21, 40.28)	20.03 (17.58, 22.17)	15.39 (12.63, 18.11)
**70-79 year**	75.63 (68.31, 81.76)	82.08 (76.04, 86.84)	69.65 (61.14, 77.05)	22.98 (20.75, 24.84)	12.00 (11.11, 12.69)	10.98 (9.64, 12.15)
**30-79 year**	**35.49 (28.97, 42.53)**	**41.59 (34.51, 49.01)**	**29.20 (23.24, 35.84)**	**126.33 (103.10, 151.39)**	**75.19 (62.4, 88.61)**	**51.14 (40.71, 62.78)**
			Central			
**30-39 year**	12.10 (8.69, 16.57)	15.93 (11.55, 21.56)	8.25 (5.82, 11.55)	6.42 (4.61, 8.79)	4.24 (3.07, 5.73)	2.18 (1.54, 3.06)
**40-49 year**	24.40 (18.34, 31.61)	31.00 (23.69, 39.35)	17.65 (12.87, 23.71)	12.45 (9.36, 16.14)	7.99 (6.11, 10.15)	4.46 (3.25, 5.99)
**50-59 year**	39.58 (31.45, 48.27)	48.29 (39.32, 57.30)	31.01 (23.69, 39.38)	23.20 (18.43, 28.29)	14.05 (11.44, 16.67)	9.15 (6.99, 11.62)
**60-69 year**	59.41 (50.75, 67.45)	67.92 (59.67, 75.07)	50.90 (41.84, 59.84)	22.19 (18.95, 25.19)	12.68 (11.14, 14.01)	9.51 (7.82, 11.18)
**70-79 year**	74.05 (66.94, 79.99)	80.61 (74.70, 85.25)	67.82 (59.57, 74.99)	16.25 (14.69, 17.55)	8.62 (7.99, 9.12)	7.63 (6.70, 8.43)
**30-79 year**	**36.26 (29.75, 43.22)**	**42.93 (35.86, 50.24)**	**29.62 (23.65, 36.23)**	**80.51 (66.04, 95.96)**	**47.57 (39.74, 55.68)**	**32.93 (26.30, 40.28)**
			West			
**30-39 year**	12.71 (9.14, 17.38)	16.63 (12.06, 22.49)	8.50 (6.00, 11.91)	7.12 (5.12, 9.74)	4.82 (3.50, 6.52)	2.30 (1.62, 3.22)
**40-49 year**	25.41 (19.11, 32.90)	32.31 (24.69, 41.00)	18.13 (13.21, 24.34)	14.68 (11.04, 19.00)	9.59 (7.33, 12.17)	5.09 (3.71, 6.84)
**50-59 year**	40.85 (32.45, 49.80)	49.90 (40.60, 59.27)	31.54 (24.08, 40.08)	24.17 (19.20, 29.47)	14.96 (12.17, 17.77)	9.21 (7.03, 11.70)
**60-69 year**	61.20 (52.28, 69.49)	70.45 (61.88, 77.89)	51.98 (42.71, 61.11)	22.50 (19.22, 25.55)	12.93 (11.36, 14.3)	9.57 (7.86, 11.25)
**70-79 year**	76.75 (69.43, 82.86)	84.35 (78.21, 89.17)	69.68 (61.25, 76.99)	16.86 (15.25, 18.20)	8.93 (8.28, 9.45)	7.93 (6.97, 8.76)
**30-79 year**	**36.83 (30.14, 44.00)**	**43.57 (36.26, 51.19)**	**29.88 (23.83, 36.60)**	**85.33 (69.84, 101.96)**	**51.24 (42.64, 60.20)**	**34.09 (27.19, 41.76)**
			Northeast			
**30-39 year**	12.97 (9.32, 17.74)	16.81 (12.20, 22.71)	9.01 (6.36, 12.61)	1.91 (1.37, 2.61)	1.26 (0.91, 1.70)	0.65 (0.46, 0.91)
**40-49 year**	25.40 (19.08, 32.94)	31.85 (24.34, 40.45)	18.79 (13.69, 25.24)	4.18 (3.14, 5.42)	2.65 (2.03, 3.37)	1.52 (1.11, 2.05)

Age group	Prevalence (%, 95% CI)			Case number (million, 95% CI)		
	**Both**	**Male**	**Female**	**Both**	**Male**	**Female**
**50-59 year**	41.77 (33.21, 50.89)	50.13 (40.86, 59.41)	33.43 (25.57, 42.39)	7.98 (6.35, 9.73)	4.79 (3.90, 5.67)	3.20 (2.45, 4.06)
**60-69 year**	61.31 (52.17, 69.87)	69.58 (60.97, 77.08)	53.53 (43.88, 63.08)	8.95 (7.62, 10.20)	4.92 (4.32, 5.46)	4.02 (3.30, 4.74)
**70-79 year**	77.32 (69.71, 83.71)	83.40 (77.23, 88.26)	72.12 (63.28, 79.81)	5.05 (4.56, 5.47)	2.51 (2.33, 2.66)	2.54 (2.23, 2.81)
**30-79 year**	**39.31 (32.25, 46.81)**	**45.52 (38.04, 53.19)**	**33.20 (26.54, 40.51)**	**26.16 (21.66, 30.82)**	**14.88 (12.57, 17.16)**	**11.29 (9.09, 13.66)**

Notes: CI, confidence interval.

Table S12. Estimated provincial prevalence and case number of carotid atherosclerosis in the mainland of China in 2020

Province	Prevalence (%, 95% CI)			Case number (million, 95% CI)		
	Both	Male	Female	Both	Male	Female
**Beijing**	37.25 (30.31, 44.78)	42.78 (35.37, 50.60)	31.54 (25.08, 38.76)	5.51 (4.48, 6.62)	3.21 (2.66, 3.80)	2.29 (1.82, 2.82)
**Fujian**	35.63 (28.94, 42.90)	44.35 (36.60, 52.55)	26.65 (21.07, 32.96)	9.14 (7.43, 11.01)	5.77 (4.76, 6.84)	3.37 (2.66, 4.17)
**Guangdong**	32.61 (26.25, 39.64)	39.06 (31.89, 46.79)	25.54 (20.07, 31.80)	23.58 (18.98, 28.66)	14.76 (12.06, 17.69)	8.81 (6.92, 10.97)
**Jiangsu**	37.83 (31.12, 44.97)	45.04 (37.73, 52.60)	30.64 (24.54, 37.37)	21.16 (17.41, 25.16)	12.58 (10.53, 14.68)	8.59 (6.88, 10.47)
**Zhejiang**	35.15 (28.65, 42.16)	41.07 (34.04, 48.44)	28.82 (22.89, 35.45)	15.04 (12.26, 18.04)	9.08 (7.53, 10.71)	5.96 (4.73, 7.33)
**Tianjin**	37.25 (30.52, 44.45)	41.99 (35.02, 49.25)	32.33 (25.85, 39.47)	3.44 (2.82, 4.11)	1.98 (1.65, 2.32)	1.47 (1.17, 1.79)
**Shanghai**	37.00 (30.36, 44.10)	42.59 (35.53, 49.94)	31.02 (24.83, 37.86)	6.35 (5.21, 7.57)	3.78 (3.15, 4.43)	2.58 (2.06, 3.14)
**Shandong**	35.93 (29.48, 42.81)	41.22 (34.45, 48.22)	30.69 (24.56, 37.45)	23.52 (19.30, 28.02)	13.43 (11.22, 15.70)	10.10 (8.08, 12.32)
**Hebei**	35.76 (29.31, 42.66)	40.79 (34.07, 47.75)	30.80 (24.62, 37.64)	16.73 (13.71, 19.96)	9.48 (7.91, 11.09)	7.25 (5.80, 8.86)
**Hainan**	31.89 (25.82, 38.53)	36.92 (30.35, 43.91)	26.37 (20.84, 32.63)	1.86 (1.50, 2.25)	1.13 (0.93, 1.34)	0.73 (0.58, 0.91)
**Anhui**	35.38 (29.07, 42.12)	41.23 (34.52, 48.16)	29.58 (23.66, 36.13)	13.15 (10.81, 15.65)	7.64 (6.39, 8.92)	5.51 (4.41, 6.73)
**Jiangxi**	35.47 (28.97, 42.45)	42.59 (35.40, 50.08)	28.31 (22.51, 34.79)	9.25 (7.56, 11.08)	5.57 (4.63, 6.55)	3.68 (2.93, 4.53)
**Hunan**	37.40 (30.73, 44.50)	44.57 (37.28, 52.09)	30.10 (24.06, 36.79)	15.48 (12.72, 18.42)	9.30 (7.78, 10.87)	6.18 (4.94, 7.55)
**Hubei**	37.50 (30.79, 44.64)	44.47 (37.17, 52.01)	30.38 (24.28, 37.11)	14.22 (11.67, 16.92)	8.52 (7.12, 9.96)	5.70 (4.55, 6.96)
**Shanxi**	35.52 (29.03, 42.48)	41.29 (34.35, 48.50)	29.51 (23.48, 36.21)	7.87 (6.43, 9.41)	4.67 (3.88, 5.48)	3.20 (2.55, 3.93)
**Henan**	35.84 (29.41, 42.70)	42.58 (35.61, 49.79)	29.44 (23.53, 35.98)	20.53 (16.85, 24.47)	11.88 (9.93, 13.89)	8.66 (6.92, 10.58)
**Guizhou**	37.97 (31.05, 45.43)	45.91 (38.11, 54.08)	29.87 (23.83, 36.61)	7.93 (6.49, 9.49)	4.85 (4.02, 5.71)	3.09 (2.46, 3.78)
**Ningxia**	33.17 (26.87, 40.05)	40.28 (33.14, 47.87)	25.85 (20.42, 32.02)	1.39 (1.13, 1.68)	0.86 (0.70, 1.02)	0.54 (0.42, 0.66)
**Inner Mongolia**	35.78 (29.16, 42.90)	42.21 (34.99, 49.74)	29.15 (23.15, 35.84)	5.94 (4.84, 7.12)	3.56 (2.95, 4.20)	2.38 (1.89, 2.93)
**Qinghai**	32.21 (25.98, 39.05)	38.21 (31.28, 45.60)	25.93 (20.43, 32.19)	1.10 (0.89, 1.34)	0.67 (0.55, 0.80)	0.43 (0.34, 0.54)
**Shaanxi**	35.72 (29.26, 42.64)	42.25 (35.21, 49.57)	29.02 (23.15, 35.53)	8.94 (7.33, 10.68)	5.36 (4.47, 6.29)	3.59 (2.86, 4.39)
**Chongqing**	36.60 (30.19, 43.40)	41.07 (34.56, 47.72)	32.12 (25.80, 39.05)	7.38 (6.09, 8.75)	4.15 (3.49, 4.82)	3.24 (2.60, 3.93)
**Sichuan**	42.14 (34.79, 49.92)	51.06 (42.90, 59.43)	33.16 (26.63, 40.34)	22.39 (18.48, 26.51)	13.61 (11.43, 15.84)	8.77 (7.05, 10.67)
**Xinjiang**	29.97 (24.06, 36.50)	34.31 (27.98, 41.12)	25.36 (19.90, 31.61)	4.40 (3.53, 5.36)	2.59 (2.11, 3.11)	1.81 (1.42, 2.25)

Province	Prevalence (%, 95% CI)			Case number (million, 95% CI)		
	Both	Male	Female	Both	Male	Female
**Gansu**	34.93 (28.57, 41.75)	41.35 (34.42, 48.54)	28.41 (22.63, 34.85)	5.35 (4.38, 6.40)	3.20 (2.66, 3.75)	2.16 (1.72, 2.65)
**Yunnan**	37.47 (30.45, 45.10)	45.11 (37.19, 53.48)	29.32 (23.25, 36.15)	10.41 (8.46, 12.53)	6.47 (5.33, 7.67)	3.94 (3.12, 4.86)
**Tibet**	26.80 (21.35, 32.92)	29.91 (24.13, 36.25)	23.30 (18.23, 29.17)	0.51 (0.40, 0.62)	0.30 (0.24, 0.36)	0.21 (0.16, 0.26)
**Guangxi**	33.49 (27.34, 40.13)	38.39 (31.85, 45.26)	28.33 (22.59, 34.73)	9.58 (7.82, 11.47)	5.63 (4.67, 6.64)	3.95 (3.15, 4.84)
**Liaoning**	41.09 (33.82, 48.77)	48.54 (40.69, 56.55)	33.79 (27.08, 41.14)	12.64 (10.41, 15.01)	7.39 (6.20, 8.62)	5.25 (4.21, 6.39)
**Heilongjiang**	37.89 (30.99, 45.24)	43.66 (36.36, 51.18)	32.17 (25.67, 39.35)	8.84 (7.23, 10.56)	5.07 (4.22, 5.95)	3.77 (3.01, 4.61)
**Jilin**	38.06 (31.14, 45.43)	42.68 (35.60, 49.96)	33.51 (26.75, 40.96)	6.59 (5.39, 7.87)	3.67 (3.06, 4.29)	2.92 (2.33, 3.57)

Notes: CI, confidence interval.

Table S13. Estimated age- and sex-specific prevalence and case number of carotid plaque by economic regions in the mainland of China in 2020

Age group	Prevalence (%, 95% CI)			Case number (million, 95% CI)		
	Both	Male	Female	Both	Male	Female
			**East**			
**30-39 year**	7.86 (6.54, 9.43)	9.47 (7.89, 11.32)	6.12 (5.07, 7.37)	7.81 (6.49, 9.36)	4.90 (4.08, 5.86)	2.91 (2.41, 3.50)
**40-49 year**	16.01 (13.53, 18.84)	19.05 (16.17, 22.30)	12.80 (10.74, 15.19)	13.11 (11.08, 15.43)	8.01 (6.80, 9.37)	5.11 (4.28, 6.06)
**50-59 year**	28.01 (24.22, 32.12)	32.59 (28.40, 37.07)	23.32 (19.96, 27.06)	23.99 (20.75, 27.52)	14.11 (12.30, 16.05)	9.88 (8.46, 11.47)
**60-69 year**	45.93 (41.13, 50.81)	51.49 (46.58, 56.38)	40.46 (35.77, 45.33)	26.95 (24.14, 29.82)	14.99 (13.56, 16.41)	11.96 (10.58, 13.41)
**70-79 year**	62.99 (58.32, 67.43)	68.22 (63.83, 72.30)	58.14 (53.22, 62.91)	19.14 (17.72, 20.49)	9.97 (9.33, 10.57)	9.17 (8.39, 9.92)
**30-79 year**	**25.57 (22.53, 28.83)**	**28.75 (25.48, 32.23)**	**22.28 (19.48, 25.32)**	**91.01 (80.18, 102.62)**	**51.98 (46.06, 58.26)**	**39.03 (34.12, 44.36)**
			**Central**			
**30-39 year**	7.51 (6.24, 9.01)	9.14 (7.61, 10.92)	5.88 (4.87, 7.08)	3.99 (3.31, 4.78)	2.43 (2.02, 2.91)	1.56 (1.29, 1.88)
**40-49 year**	15.70 (13.27, 18.47)	18.77 (15.94, 21.96)	12.57 (10.55, 14.92)	8.02 (6.77, 9.43)	4.84 (4.11, 5.66)	3.18 (2.66, 3.77)
**50-59 year**	27.25 (23.57, 31.26)	31.91 (27.81, 36.30)	22.66 (19.39, 26.30)	15.97 (13.81, 18.32)	9.28 (8.09, 10.56)	6.69 (5.72, 7.76)
**60-69 year**	45.29 (40.59, 50.06)	50.97 (46.13, 55.76)	39.62 (35.05, 44.36)	16.91 (15.16, 18.70)	9.51 (8.61, 10.41)	7.40 (6.55, 8.29)
**70-79 year**	61.73 (57.18, 66.05)	67.07 (62.77, 71.07)	56.64 (51.87, 61.27)	13.54 (12.55, 14.49)	7.17 (6.71, 7.60)	6.37 (5.83, 6.89)
**30-79 year**	**26.32 (23.24, 29.60)**	**29.99 (26.66, 33.51)**	**22.66 (19.84, 25.71)**	**58.43 (51.6, 65.72)**	**33.24 (29.55, 37.14)**	**25.19 (22.06, 28.58)**
			**West**			
**30-39 year**	7.86 (6.54, 9.42)	9.54 (7.95, 11.41)	6.06 (5.02, 7.30)	4.41 (3.66, 5.28)	2.77 (2.31, 3.31)	1.64 (1.36, 1.97)
**40-49 year**	16.33 (13.80, 19.20)	19.56 (16.61, 22.89)	12.91 (10.83, 15.31)	9.43 (7.97, 11.09)	5.80 (4.93, 6.79)	3.63 (3.04, 4.30)
**50-59 year**	28.06 (24.26, 32.19)	32.94 (28.69, 37.49)	23.04 (19.71, 26.75)	16.60 (14.35, 19.04)	9.88 (8.6, 11.24)	6.72 (5.75, 7.81)
**60-69 year**	46.63 (41.79, 51.55)	52.84 (47.82, 57.82)	40.44 (35.78, 45.29)	17.14 (15.36, 18.95)	9.70 (8.78, 10.61)	7.45 (6.59, 8.34)
**70-79 year**	64.04 (59.35, 68.50)	70.27 (65.79, 74.44)	58.24 (53.36, 62.98)	14.07 (13.04, 15.05)	7.44 (6.97, 7.88)	6.63 (6.07, 7.17)
**30-79 year**	**26.61 (23.47, 29.96)**	**30.26 (26.86, 33.87)**	**22.84 (19.99, 25.93)**	**61.65 (54.39, 69.42)**	**35.59 (31.58, 39.84)**	**26.06 (22.81, 29.58)**
			**Northeast**			
**30-39 year**	8.06 (6.70, 9.66)	9.65 (8.04, 11.53)	6.42 (5.32, 7.73)	1.19 (0.99, 1.42)	0.72 (0.60, 0.86)	0.46 (0.39, 0.56)
**40-49 year**	16.37 (13.83, 19.26)	19.28 (16.37, 22.56)	13.38 (11.23, 15.88)	2.69 (2.27, 3.17)	1.60 (1.36, 1.88)	1.09 (0.91, 1.29)

Age group	Prevalence (%, 95% CI)			Case number (million, 95% CI)		
	Both	Male	Female	Both	Male	Female
**50-59 year**	28.81 (24.93, 33.04)	33.18 (28.93, 37.73)	24.45 (20.93, 28.36)	5.51 (4.77, 6.32)	3.17 (2.76, 3.60)	2.34 (2.00, 2.71)
**60-69 year**	46.61 (41.69, 51.61)	51.97 (46.98, 56.94)	41.55 (36.72, 46.59)	6.80 (6.09, 7.53)	3.68 (3.32, 4.03)	3.12 (2.76, 3.50)
**70-79 year**	64.37 (59.55, 68.96)	69.29 (64.81, 73.46)	60.16 (55.06, 65.11)	4.21 (3.89, 4.51)	2.09 (1.95, 2.21)	2.12 (1.94, 2.29)
**30-79 year**	**28.56 (25.21, 32.13)**	**31.77 (28.23, 35.51)**	**25.39 (22.24, 28.80)**	**19.21 (17.02, 21.52)**	**10.54 (9.40, 11.73)**	**8.67 (7.61, 9.80)**

Notes: CI, confidence interval.

Table S14. Estimated provincial prevalence and case number of carotid plaque in the mainland of China in 2020

	Prevalence (%, 95% CI)			Case number (million, 95% CI)		
	Both	Male	Female	Both	Male	Female
**Beijing**	26.99 (23.74, 30.48)	29.76 (26.32, 33.43)	24.12 (21.08, 27.43)	3.99 (3.51, 4.50)	2.24 (1.98, 2.51)	1.75 (1.53, 1.99)
**Fujian**	25.19 (22.14, 28.48)	29.99 (26.51, 33.72)	20.24 (17.64, 23.09)	6.46 (5.68, 7.31)	3.90 (3.45, 4.39)	2.56 (2.23, 2.92)
**Guangdong**	22.99 (20.12, 26.10)	26.35 (23.18, 29.77)	19.30 (16.77, 22.07)	16.62 (14.55, 18.87)	9.96 (8.76, 11.25)	6.66 (5.78, 7.61)
**Jiangsu**	27.55 (24.36, 30.94)	31.61 (28.14, 35.27)	23.49 (20.60, 26.62)	15.41 (13.63, 17.31)	8.83 (7.86, 9.85)	6.58 (5.77, 7.46)
**Zhejiang**	25.25 (22.23, 28.49)	28.33 (25.09, 31.78)	21.94 (19.16, 24.97)	10.80 (9.51, 12.19)	6.27 (5.55, 7.03)	4.54 (3.96, 5.16)
**Tianjin**	27.05 (23.87, 30.44)	29.26 (25.98, 32.72)	24.75 (21.68, 28.07)	2.50 (2.21, 2.81)	1.38 (1.22, 1.54)	1.12 (0.98, 1.27)
**Shanghai**	26.85 (23.71, 30.19)	29.72 (26.41, 33.22)	23.78 (20.83, 26.95)	4.61 (4.07, 5.18)	2.64 (2.34, 2.95)	1.97 (1.73, 2.24)
**Shandong**	26.15 (23.10, 29.41)	28.82 (25.62, 32.20)	23.51 (20.60, 26.65)	17.12 (15.12, 19.25)	9.39 (8.35, 10.49)	7.73 (6.78, 8.77)
**Hebei**	26.02 (22.97, 29.27)	28.49 (25.32, 31.84)	23.57 (20.64, 26.74)	12.17 (10.74, 13.69)	6.62 (5.88, 7.40)	5.55 (4.86, 6.30)
**Hainan**	22.70 (19.92, 25.71)	25.15 (22.19, 28.33)	20.01 (17.44, 22.82)	1.32 (1.16, 1.50)	0.77 (0.68, 0.86)	0.56 (0.48, 0.63)
**Anhui**	25.79 (22.80, 28.97)	28.93 (25.75, 32.27)	22.67 (19.86, 25.69)	9.58 (8.47, 10.77)	5.36 (4.77, 5.98)	4.22 (3.70, 4.79)
**Jiangxi**	25.56 (22.52, 28.81)	29.51 (26.17, 33.06)	21.58 (18.85, 24.53)	6.67 (5.88, 7.52)	3.86 (3.42, 4.32)	2.81 (2.45, 3.19)
**Hunan**	27.15 (24.00, 30.52)	31.20 (27.75, 34.84)	23.04 (20.18, 26.13)	11.24 (9.94, 12.64)	6.51 (5.79, 7.27)	4.73 (4.15, 5.37)
**Hubei**	27.21 (24.04, 30.59)	31.08 (27.63, 34.73)	23.25 (20.36, 26.37)	10.31 (9.11, 11.60)	5.95 (5.29, 6.65)	4.36 (3.82, 4.94)
**Shanxi**	25.63 (22.59, 28.89)	28.64 (25.40, 32.07)	22.50 (19.67, 25.57)	5.68 (5.01, 6.40)	3.24 (2.87, 3.63)	2.44 (2.13, 2.78)
**Henan**	26.08 (23.04, 29.32)	29.81 (26.52, 33.29)	22.54 (19.74, 25.56)	14.94 (13.20, 16.80)	8.32 (7.40, 9.29)	6.63 (5.80, 7.52)
**Guizhou**	27.36 (24.13, 30.82)	31.78 (28.18, 35.61)	22.84 (19.99, 25.93)	5.72 (5.04, 6.44)	3.36 (2.97, 3.76)	2.36 (2.07, 2.68)
**Ningxia**	23.60 (20.72, 26.71)	27.48 (24.25, 30.94)	19.61 (17.08, 22.37)	0.99 (0.87, 1.12)	0.58 (0.52, 0.66)	0.41 (0.35, 0.46)
**Inner Mongolia**	25.69 (22.61, 29.00)	29.10 (25.75, 32.65)	22.18 (19.36, 25.23)	4.27 (3.75, 4.82)	2.45 (2.17, 2.75)	1.81 (1.58, 2.06)
**Qinghai**	22.81 (19.98, 25.88)	25.85 (22.75, 29.18)	19.63 (17.08, 22.42)	0.78 (0.69, 0.89)	0.45 (0.40, 0.51)	0.33 (0.29, 0.38)
**Shaanxi**	25.84 (22.81, 29.09)	29.41 (26.11, 32.90)	22.18 (19.41, 25.18)	6.47 (5.71, 7.28)	3.73 (3.31, 4.17)	2.74 (2.40, 3.11)
**Chongqing**	26.87 (23.80, 30.13)	29.04 (25.91, 32.32)	24.69 (21.68, 27.94)	5.42 (4.80, 6.08)	2.93 (2.62, 3.27)	2.49 (2.18, 2.81)
**Sichuan**	30.77 (27.26, 34.50)	36.01 (32.11, 40.11)	25.49 (22.38, 28.84)	16.34 (14.48, 18.32)	9.60 (8.56, 10.69)	6.74 (5.92, 7.63)
**Xinjiang**	21.17 (18.51, 24.06)	23.08 (20.28, 26.10)	19.14 (16.63, 21.90)	3.11 (2.72, 3.53)	1.74 (1.53, 1.97)	1.36 (1.18, 1.56)

	Prevalence (%, 95% CI)			Case number (million, 95% CI)		
	Both	Male	Female	Both	Male	Female
**Gansu**	25.23 (22.25, 28.42)	28.71 (25.48, 32.14)	21.69 (18.97, 24.64)	3.87 (3.41, 4.36)	2.22 (1.97, 2.48)	1.65 (1.44, 1.87)
**Yunnan**	26.73 (23.50, 30.21)	30.87 (27.27, 34.72)	22.31 (19.47, 25.40)	7.42 (6.53, 8.39)	4.43 (3.91, 4.98)	3.00 (2.62, 3.41)
**Tibet**	18.74 (16.33, 21.39)	19.80 (17.31, 22.51)	17.55 (15.23, 20.12)	0.35 (0.31, 0.40)	0.20 (0.17, 0.23)	0.16 (0.14, 0.18)
**Guangxi**	24.16 (21.30, 27.24)	26.54 (23.52, 29.75)	21.66 (18.95, 24.59)	6.91 (6.09, 7.79)	3.89 (3.45, 4.36)	3.02 (2.64, 3.42)
**Liaoning**	29.94 (26.47, 33.63)	34.06 (30.31, 38.01)	25.90 (22.70, 29.34)	9.21 (8.14, 10.35)	5.19 (4.62, 5.79)	4.02 (3.53, 4.56)
**Heilongjiang**	27.42 (24.17, 30.90)	30.30 (26.87, 33.93)	24.57 (21.50, 27.89)	6.40 (5.64, 7.21)	3.52 (3.12, 3.94)	2.88 (2.52, 3.27)
**Jilin**	27.64 (24.37, 31.13)	29.70 (26.36, 33.23)	25.60 (22.41, 29.06)	4.79 (4.22, 5.39)	2.55 (2.27, 2.86)	2.23 (1.95, 2.53)

Notes: CI, confidence interval.

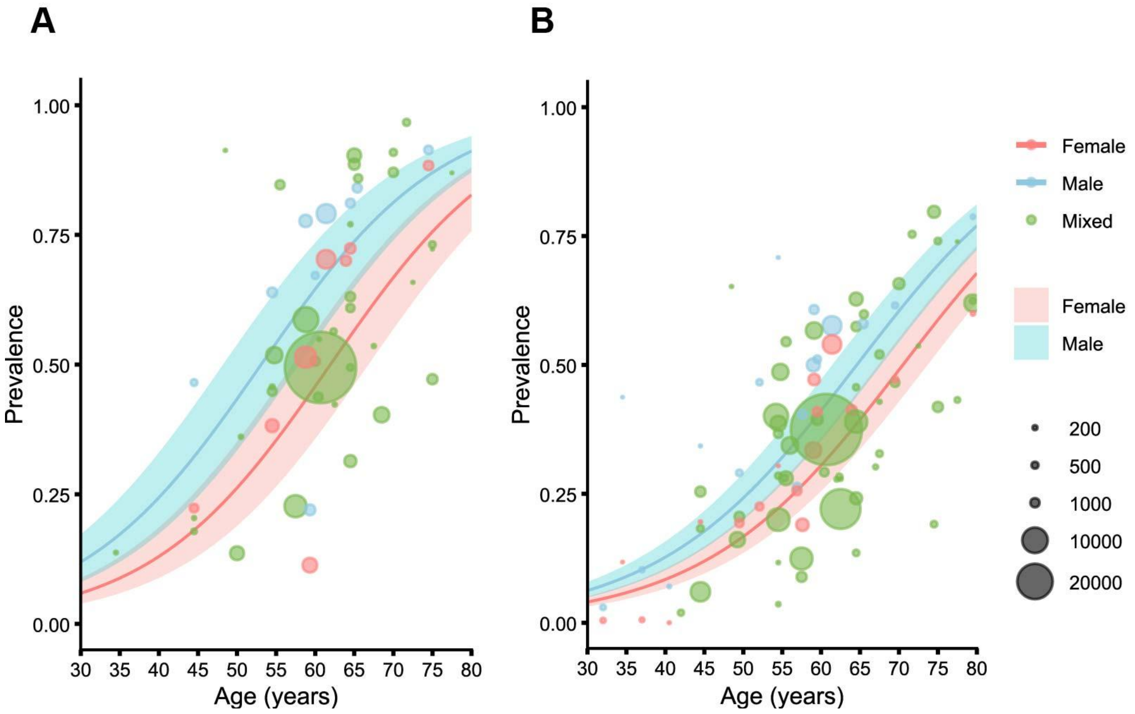

Figure S1. Multilevel mixed-effects meta-regression models for prevalence patterns of the prevalence of carotid atherosclerosis and carotid plaque

Notes: (A) Carotid atherosclerosis; (B) Carotid plaque.

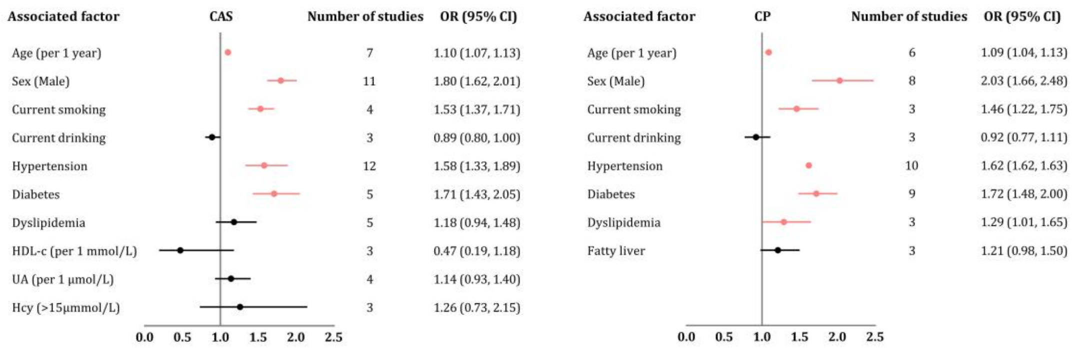

Figure S2. Summary of associated factors of carotid atherosclerosis and carotid plaque

Notes: CAS, carotid atherosclerosis; CP, carotid plaque; HDL-c, high-density lipoprotein cholesterol; UA, uric acid; Hcy, homocysteine; OR, odds ratio; CI, confidence interval.

Appendix 4. Full list of the included articles (n=82)

Article ID	Reference
**A01**	Fan Jingwen, Yang Yongli, Jia Xiaocan, et al. Metabolic score and its components are associated with carotid plaque prevalence in young adults. [J] Endocrine. 2024.
**A02**	Fu Jingzhu, Deng Yuhan, Ma Yuan, et al. National and Provincial-Level Prevalence and Risk Factors of Carotid Atherosclerosis in Chinese Adults. [J] JAMA network open. 2024,7(1):e23.
**A03**	Wang Yuanping, Chen Cheng, Lin Qiaofen, et al. The ratio of systolic and diastolic pressure is associated with carotid and femoral atherosclerosis. [J] Frontiers in Cardiovascular Medicine. 2024,11:1353945.
**A04**	Yong Yufei, Dong Hui, Zhu Yan, et al. The association between hyperhomocysteinemia and the prevalence of bilateral carotid atherosclerotic plaques in a middle-aged population. [J] Clinical neurology and neurosurgery. 2024.
**A05**	Yu Jiayuan, Zhao Di, Jiang Xin, et al. (于佳园, 赵娣, 姜新, 等) Association analysis on peripheral blood lymphocyte subsetsand occurrence of carotid atherosclerosis. (外周血淋巴细胞亚群与颈动脉粥样硬化发生的关联性分析) [J] Journal of Jilin University (Medicine Edition). (吉林大学学报(医学版).) 2024,50(05).
**A06**	Zhang Xue. (张雪) Ultrasonic investigation of carotid artery and lower extremity atherosclerosis in the elderly population in a community in Beijing. (超声对北京市某社区老年人群颈动脉和下肢动脉粥样硬化的调查研究) [J] Journal of Imaging Research and Medical Applications. (影像研究与医学应用) 2024,8(6).
**A07**	Zhou Naqi. (周娜麒) Relationship between overweight and obesity and carotid atherosclerotic in physical examination population. (体检人群超重和肥胖与颈动脉粥样硬化的关系) [D] Inner Mongolia Medical University. (内蒙古医科大学) 2024.
**A08**	Jiang Peng. (江鹏) Epidemiological investigation of carotid plaque in residents in Mengyang Town, Pengzhou City. (彭州市濛阳镇居民颈动脉斑块的流行病学调查) [D] Chengdu Medical College. (成都医学院) 2023.
**A09**	Liu Chunxing, Yang Xiaolong, Ji Mengmeng, et al. Sex-specific association between carotid atherosclerosis and fundus arteriosclerosis in a Chinese population: a retrospective cross-sectional study. [J] European journal of medical research. 2023,28(1):518.
**A10**	Liu Dongjie. (刘冬杰) Analysis of influencing factors of subclinical carotid atherosclerosis in healthy physical examination population. (健康体检人群亚临床颈动脉粥样硬化的影响因素分析) [D] Dalian Medical University. (大连医科大学) 2023.
**A11**	Pan Jia, Yang Bo, Wang Zihang, et al. Triglyceride-Glucose Index is Related to Carotid Artery Plaque in Railway Workers: A Cross-Sectional Study. [J] Diabetes, Metabolic Syndrome and Obesity. 2023,16:2561-2571.
**A12**	Tao Lijun, Chen Runlin, He Tufeng, et al. (陶俐均, 陈润霖, 何土凤, 等) Risk factors and predictive model of carotid plaque in population at low risk of cardiovascular disease. (心血管疾病低风险群体颈动脉斑块危险因素及预测模型构建) [J] China Preventive Medicine. (中国预防医学杂志) 2023,24(9).
**A13**	Wang Shuwei, Chen Jin, Ding Chen, et al. Utilizing machine learning algorithms for the prediction of carotid artery plaques in a Chinese population. [J] Frontiers in Physiology. 2023,14.
**A14**	Yang Tingting, Wang Yating, Zhang Xiaoke, et al. Prevalence and influencing factors of abnormal carotid artery intima-media thickness in Henan Province in China. [J] Frontiers in Endocrinology. 2023,14:1266207.
**A15**	Yu Putian. (于溥田) Investigation on the prevalence of carotid atherosclerosis and the control of risk factors among the elderly in the community under the background of family physician contract. (家庭医生签约背景下社区老年人颈动脉粥样硬化患病率及其危险因素控制情况调查) [D] China Medical University. (中国医科大学) 2023.
**A16**	Zeng Nimei, Shen Yu’e, Li Yuan, et al. Association between remnant cholesterol and subclinical carotid atherosclerosis among Chinese general population in health examination. [J] Journal of stroke and cerebrovascular diseases : the official journal of National Stroke Association. 2023,32(8):107234.
**A17**	Shen Qiuyu, Zheng Jinke, Gao Jie, et al. (沈秋育, 郑进科, 高捷, 等) Investigation and Analysis of Carotid Artery Ultrasonography in Civil Aviation Pilots. (民航飞行员颈动脉超声检查情况调查分析) [J] Journal of Aerospace Medicine. (航空航天医学杂志) 2022,33(8).

Article ID	Reference
**A18**	Wang Anran. (王安然) The prevalence of carotid atherosclerosis, associated risk factors, and its relationship with incident cardiovascular disease in middle-aged and older adults. (中老年人群颈动脉粥样硬化患病现状、危险因素及与新发心血管疾病的关联研究) [D] Zhengzhou University. (郑州大学) 2022.
**A19**	Zhu Lei. (朱蕾) Study on the Effectiveness of Carotid Ultrasonography in Health Checkups. (颈动脉彩超在健康体检中的效果研究) [J] Our Health. (健康之友) 2022(13).
**A20**	Dai Wen, Sun Zhenwei, Zheng Xican. (代稳, 孙振威, 郑喜灿) Correlation between Carotid Plaque and Multiple Metabolic Abnormalities in Healthy People. (健康体检人群颈动脉斑块与多代谢异常检出现况的相关性研究) [J] World Latest Medicine Information. (世界最新医学信息文摘) 2021,21(4).
**A21**	H. Shu-xia. (黄署霞) Analysis of carotid artery ultrasonography in college retired elderly people. (某高校退休老人颈动脉超声检查分析) [J] Chinese Journal of School Doctor. (中国校医) 2021,35(1).
**A22**	He Zhili, Liu Jianfang, Xiong Yan. (贺志力, 刘建芳, 熊燕) Investigation of Traditional Chinese Medicine Constitution in Patients with Carotid Plaques in Nanchang City. (南昌市颈动脉斑块患者中医体质调查) [J] Guangming Journal of Chinese Medicine. (光明中医) 2021,36(22).
**A23**	Huang Yuqing, Liu Lin, Yu Yuling, et al. The relationship between famine exposure during early life and carotid plaque in adulthood. [J] European journal of clinical nutrition. 2021,75(3):546-554.
**A24**	Liu Fang, Wang Zheng, Cao Xia, et al. Relationship between small dense low-density lipoprotein cholesterol with carotid plaque in Chinese individuals with abnormal carotid artery intima-media thickness. [J] BMC cardiovascular disorders. 2021,21(1):216.
**A25**	Lu Yu. (卢瑜) Correlation study of diet and lifestyle with carotid artery plaque formation in health people in Shenzhen. (深圳地区健康体检人群饮食生活方式与颈动脉斑块形成的相关性研究) [D] Guangxi Medical University. (广西医科大学) 2018,34(10).
**A26**	Shen Zhiyuan, Jin Haiqiang, Peng Qing, et al. Co-existence and interrelationship between intracranial artery stenosis and extracranial carotid atherosclerosis in an asymptomatic rural population of 13 villages in northern China. [J] Clinical neurology and neurosurgery. 2021,210:107013.
**A27**	Tang Qingwu, Wang Qingfei, Zhu Xiangbao. (唐庆武,王青飞,朱相宝) Study on the application value of carotid artery ultrasound in screening high-risk population of stroke. (颈动脉超声检测在脑卒中高危人群筛查中的应用价值研究) [J] China Modern Medicine. (中国当代医药) 2021,28(13).
**A28**	Wu Tzuwei, Chou Chaoliang, Cheng Chunfang, et al. Prevalences of diabetes mellitus and carotid atherosclerosis and their relationships in middle-aged adults and elders: a community-based study. [J] Journal of the Formosan Medical Association. 2022,121(6):1133-1140.
**A29**	Xing Liying, Li Ru, Zhang Suli, et al. High Burden of Carotid Atherosclerosis in Rural Northeast China: A Population-Based Study. [J] Frontiers in Neurology. 2021. 12: p. 597992.
**A30**	Yu Y, Zhang F.-L, Yan X.-L., et al. Visceral adiposity index and cervical arterial atherosclerosis in northeast China: a population based cross-sectional survey. [J] European journal of neurology. 2021,28(1):161-171.
**A31**	Zhang Jie. (张洁) Prevalence and risk factors of carotid plaque in rural China . (中国农村地区颈动脉斑块的患病率及其相关危险因素) [D] Shandong First Medical University. (山东第一医科大学) 2021.
**A32**	Zhang Nan, Pan Xiaofang, Jia Xiaodong, et al. (张楠, 潘晓芳, 贾晓东, 等) Correlation study between carotid atherosclerotic plaque formation and TCM syndrome factors in people with normal blood lipid. (脂质蓄积指数在大连地区女性人群颈动脉硬化筛查中的价值) [J] Chinese Journal of Postgraduates of Medicine. (中国医师进修杂志) 2021,44(1).
**A33**	Guo Liping, Kang Rui. (郭丽萍,康瑞) Prevalence of cervical arteriosclerosis and related factors among aerospace Staff in Beijing. (北京地区航天系统职工颈动脉粥样硬化患病情况及相关因素分析) [J] Chinese and Foreign Medical Research. (中外医学研究) 2020,18(23):175-177.
**A34**	He Miao, Guo Zaogeng, Lu Zuxun, et al. High milk consumption is associated with carotid atherosclerosis in middle and old-aged Chinese. [J] International Journal of Cardiology: Hypertension. 2020.5.
**A35**	Huang Zhixin, Chen Lihua, Xiong Ran, et al. Essen Stroke Risk Score Predicts Carotid Atherosclerosis in Chinese Community Populations. [J] Risk management and healthcare policy. 2020. 13: p. 2115-2123.
**A36**	Liang Jun, He Daikun, Liu Yang. (梁君, 何岱昆, 刘洋) Analysis of carotid plaque detection rate and risk factors in male and female at different ages. (不同年龄阶段男女性颈动脉斑块检出率及危险因素分析) [J] Chinese Primary Health Care. (中国初级卫生保健) 2020,34(10).
**A37**	Ma Shouyuan, Wang Shuxia, Liu Jianfeng, et al. (马守原, 王曙霞, 刘剑锋, 等) Association between hypertension and carotid plaque in a physical examination population: a cross-sectional study. (某体检人群血压水平与颈动脉斑块相关性的横断面研究) [J] Chinese Journal of Health Management. (中华健康管理学杂志) 2020,14(1).

Article ID	Reference
**A38**	Song Yang, Fan Xuesong, Wang Enshi, et al. (宋扬, 范雪松, 王恩世, 等) Study on the relationship between serum apolipoprotein E and carotid atherosclerotic plaque. (血清载脂蛋白 E 水平与颈动脉粥样硬化斑块的关系研究) [J] Laboratory Medicine and Clinic. (检验医学与临床) 2020,17(15).
**A39**	Yang Ying, Fan Fangfang, Gao Lan, et al. The relationship between carotid intima-media thickness and carotid plaque: a cohort study in China. [J] Journal of Human Hypertension. 2020,34(6):468-473.
**A40**	Yuan Qinghong. (袁清红) The Clinical Value of Carotid Ultrasonography in Health Examination Populations. (颈动脉彩超检查在体检人群中应用价值) [J] Consume guide. (消费导刊) 2020(44).
**A41**	Fang Jianfei, Li Yawen. (方建飞, 李亚文) Analysis of Factors Associated with Carotid Intima-Media Thickening and Plaque Formation. (颈动脉内中膜增厚和斑块形成的相关因素分析) [J] Modern Practical Medicine. (现代实用医学) 2019,31(2).
**A42**	Lin Yanhua, Zhu Yongjian, Jia Wenrui, et al. Association between lipid profiles and presence of carotid plaque. [J] Scientific reports. 2019,9(1):18011.
**A43**	Wei Qiong'e, Xiong Xiang, Ling Chenji, et al. (魏琼娥, 熊祥, 玲沈继, 等) Investigation of Carotid Artery Status Among Faculty and Staff of a University in Yunnan Province. (云南省某高校教职工颈动脉情况调查) [J] Health for Everyone. (人人健康) 2021,21(395).
**A44**	Xiao Meifang, Xie Sisi, Wang Baoxiang, et al. (萧梅芳, 谢思思, 王保祥, 等) Investigation on carotid atherosclerosis and risk factors in normal physical examination crowd. (体检人群颈动脉粥样硬化筛查及危险因素分析) [J] China Journal of Modern Medicine. (中国现代医学杂志) 2019,29(18).
**A45**	Zhou Pingan, Zhang Chenhuan, Chen Yanru, et al. Association between Metabolic Syndrome and Carotid Atherosclerosis: A Cross-sectional Study in Northern China. [J] Biomedical and environmental sciences: BES. 2019,32(12):914-921.
**A46**	Chen Huan. (陈焕) The relationship between atherogenic index of plasma and other traditional blood lipid parameters and carotid atherosclerosis. (血浆致动脉硬化指数及传统血脂指标与颈动脉粥样硬化的关系比较) [D] Nanhua University. (南华大学) 2018.
**A47**	Fan Xuesong, Wang Enshi, He Jianxun, et al. (范雪松, 王恩世, 贺建勋, 等) Small dense low-density lipoprotein cholesterol and sdLDL-C/LDL-C ratio associate with carotid atherosclerotic plaque. (小而密低密度脂蛋白胆固醇及其与低密度脂蛋白胆固醇之比与颈动脉粥样硬化斑块的关系) [J] Chinese Journal of Laboratory Medicine. (中华检验医学杂志) 2018,41(3).
**A48**	Lin Chengguo, Sun Xiaohui, Lin Hanli, et al. Association between hyperhomocysteinemia and metabolic syndrome with early carotid artery atherosclerosis: A cross-sectional study in middle-aged Chinese population. [J] Nutrition (Burbank, Los Angeles County, Calif.). 2018,53: 115-119.
**A49**	Liu Qingxiang, Chen Shengyun, Liu Yanfang, et al. (刘清香, 陈胜云, 刘艳芳, 等) Association between Ideal Cardiovascular Health Index and Carotid Arthrosclerosis. (理想心血管健康指标与颈动脉粥样硬化的关系研究) [J] Chinese Journal of Stroke. (中国卒中杂志) 2018,13(7).
**A50**	Shi Min. (史敏) Analysis of carotid plaque stability and related influencing factors - a population-based study. (颈动脉斑块稳定性及相关影响因素分析-一项基于人群的研究) [D] Tianjin Medical University. (天津医科大学) 2018.
**A51**	Tian Jing. (田晶) Correlation between brachial ankle pulse wave velocity and carotid intima media thickness in middle-aged and elderly patients. (中老年臂踝脉搏波传导速度与颈动脉内膜中膜厚度的相关性研究) [D] Chongqing Medical University. (重庆医科大学) 2018.
**A52**	Weng Genlong, Xu Yifen, Lu Jianlin. (翁根龙, 徐怡芬, 陆建林) Risk factors of carotid atherosclerosis among community elderly in Suzhou city: a cross-sectional study. (社区老年人群颈动脉粥样硬化危险因素调查) [J] Chinese Journal of Public Health. (中国公共卫生) 2018. 34(10).
**A53**	Xia Ronghui, Nie Ying, Zhao Miansong, et al. (夏蓉晖, 聂颖, 赵绵松, 等) Current prevalence of carotid artery atherosclerosis and risk factors among retired workers in a university. (某高校退休职工颈动脉粥样硬化现状调查及危险因素分析) [J] Military Medical Sciences. (军事医学) 2018,42(5).
**A54**	Yang Yingxia, Yang Rong, Liu Zhijuan. (杨映霞, 杨蓉, 刘志娟) Analysis of risk factors of subclinical carotid atherosclerosis in middle-aged and elderly people in Dingxi city. (定西市中老年人群亚临床期颈动脉硬化的危险因素分析) [J] Chinese Journal of Public Health Engineering. (中国卫生工程学) 2018,17(3).
**A55**	You Kai, Zhao Hongye, Li Changqin, et al. (游凯, 赵红叶, 李长青, 等) Analysis of carotid ultrasound screening results of 2445 residents aged ≥ 50 years in Shunyi District, Beijing. (北京市顺义区 2 445 名年龄≥50 岁居民颈动脉超声筛查结果分析) [J] Chinese Journal of Cerebrovascular Diseases. (中国脑血管病杂志) 2018,15(8).
**A56**	Zhang Benna, Jia Jia, Liu Zhike, et al. (张本娜, 贾佳, 刘志科, 等) Correlation between plaques at different sites in the carotid arteries and risks of coronary heart disease. (颈动脉不同部位斑块与冠心病的关联研究) [J] Journal of third military medical university. (第三军医大学学报) 2018,40(2).

Article ID	Reference
A57	Fan Fengjuan, Huang Xiaona. (范凤娟, 黄晓娜) Doppler ultrasound screening and risk factor analysis of carotid atherosclerosis in elderly residents. (老年居民颈动脉硬化多普勒超声筛查及其危险因素分析) [J] Chinese Journal of Public Health Engineering. (中国卫生工程学) 2017,16(4).
A58	Li Xiufeng, Xu Xu, Liang Guowei, et al. (李秀峰, 徐旭, 梁国威, 等) Association between small dense low-density lipoprotein cholesterol and carotid atherosclerosis. (血清小而密低密度脂蛋白胆固醇与颈动脉粥样硬化的相关性) [J] Chinese Medical Journal. (中华医学杂志) 2017,97(48):3802-3805.
A59	Lu Jiqiang, Shen Zhenhai, Lu Yun, et al. (陆继强, 沈振海, 陆昀, 等) Detection rate and risk factors of carotid plaque formation in healthy population. (健康人群颈动脉斑块形成的检出率及其危险因素) [J] Hainan Medical Journal. (海南医学) 2017,28(5).
A60	Robert Clarke, Du Huaidong, Om Kurmi, et al. Burden of carotid artery atherosclerosis in Chinese adults: Implications for future risk of cardiovascular diseases. [J] European journal of preventive cardiology. 2017,24(6):647-656.
A61	Shang Jing, Li Wei, Xu Fuyin, et al. (商静, 李玮, 徐付印, 等) Carotid atherosclerotic lesions detected with ultrasonography in the population ≥ 45 years of age in dongying area. (超声评价东营地区 45 岁及以上人群颈动脉粥样硬化现状) [J] Chinese Journal of Arteriosclerosis. (中国动脉硬化杂志) 2017,25(3).
A62	Wang Xiujuan, He Wen, Li Jinhe, et al. (王秀娟, 贺文, 李金和, 等) The value of carotid color Doppler ultrasound in physical examination. (颈动脉彩超在健康体检中的价值研究) [J] Journal of Imaging Research and Medical Applications. (影像研究与医学应用) 2018,1(16).
A63	Zhang Lei, Du Zhixing, Jiao Liya, et al. (张磊, 杜志兴, 焦丽亚, 等) Prevalence of Carotid Plaques and Analysis of Related Risk Factors Among Individuals Aged 40 and Above Undergoing Health Examinations in Shijiazhuang City. (石家庄市 40 岁以上体检人群颈动脉斑块患病率及相关危险因素分析) [J] Journal of Hebei Medical University. (河北医科大学学报) 2017,38(2).
A64	Zhang Yong. (张勇) The Association between Ideal Cardiovascular Health Metrics and Carotid Intima-Media Thickness. (理想心血管健康与颈动脉内中膜厚度的相关性研究) [D] Inner Mongolia Medical University. (内蒙古医科大学) 2017.
A65	Zhao Xiaoxia, Zhang Yinxia, Gao Jing, et al. (赵晓霞, 张银霞, 高静, 等) Study on the incidence and influencing factors of carotid atherosclerosis in middle-aged and elderly teachers in colleges and universities by ultrasound screening. (超声筛查高校中老年教师颈动脉粥样硬化发病情况及其影响因素研究) [J] Journal of Northwest University for Nationalities (Natural Science). (西北民族大学学报（自然科学版）) 2017,38(4).
A66	Chen Xun, Li Guohui, Huang Yunqi, et.al. (陈绚, 李国辉, 黄云旗, 等) Role of carotid artery sonography in screening for high-risk factors of carotid stenosis and stroke. (颈动脉超声应用筛查颈动脉狭窄及脑卒中高危因素分析) [J] Journal of International Neurology and Neurosurgery. (国际神经病学神经外科学杂志) 2016, 43(4):302-305.
A67	Xia Ting, Li Juansheng, Pu Hongquan, et al. (夏婷, 李娟生, 蒲宏全, 等) Correlation between metabolic syndrome and carotid plaque of middle aged and aged people and its gender difference. (中老年人群代谢综合征与颈动脉斑块的关联性及其性别差异研究) [J] Chinese General Practice. (中国全科医学) 2016, 19(28):3422-3427.
A68	Zhang Yuma. (张玉马) Study on prevalence of CAS and relevant factors in Briat Mongolian and Han population. (布里亚特蒙古族与汉族人群颈动脉粥样硬化患病率及相关因素调查研究分析) [D] Inner Mongolia University for Nationalities. (内蒙古民族大学) 2016.
A69	Zhao Wei, Fan Chunqiu. (赵伟, 樊春秋) Risk factors of carotid atherosclerosis in the community aged over 50 years. (社区 50 岁以上人群颈动脉硬化危险因素调查分析) [J] Chinese Journal of Medicinal Guide. (中国医药导刊) 2016, 18(3).
A70	Zhao Wei, Wu Yanan, Shi Min, et.al. Sex Differences in Prevalence of and Risk Factors for Carotid Plaque among Adults: a population-based cross-sectional study in rural China. [J] Scientific Reports. 2016, 6:38618.
A71	Gao Yu, Zhang Minyu, Rong Hui, et.al. (高宇, 张敏郁, 荣辉,等) Results of carotid ultrasound screening in stroke high risk population. (颈动脉超声筛查脑卒中高危人群结果分析) [J] Chinese Journal of Evidence-Bases Cardiovascular Medicine. (中国循证心血管医学杂志) 2014(6):690-692.
A72	Guo Qiuxiang, Lvjuan, Liwei, et.al. (郭秋香, 吕娟, 李伟,等) Screening for carotid atherosclerosis in people aged 55-75 years and comparison with results in Beijing. (超声筛查包头市 55-75 岁颈动脉粥样硬化病变与北京相应人群对比情况) [J] China Health Care & Nutrition. (中国保健营养旬刊) 2014(1):484-484.
A73	Lu Lu, Gao Zhengnan. (芦鹭, 高政南) An analysis of risk factors for carotid intima-media thickness in middle and older people in a community, Dalian. (大连社区中老年人群颈动脉内膜厚度相关危险因素分析) [J] Journal of Practical Diabetology. (实用糖尿病杂志) 2014(2):58-59.
A74	Ma Yifei. (马亦飞) An investigation of carotid atherosclerosis detected with ultrasonography and its influencing factors in elderly dwellers in rural areas. (农村老年居民颈动脉硬化超声检查及其影响因素调查) [J] Chinese Journal of Public Health Management. (中国公共卫生管理) 2014(6):856-857.

Article ID	Reference
**A75**	Wang Li. (王立) The research of elevated homocysteine levels and carotid atherosclerosis in a rural population lvliang Shanxi. (山西吕梁农村人群血清同型半胱氨酸与颈动脉粥样硬化相关性研究) [J] Shanxi Medical University. (山西医科大学) 2014.
**A76**	Liu Xue. (柳雪) Postprandial blood glucose fluctuation and carotid artery intima-media thickness in correlation analysis. (餐后血糖波动与颈动脉内膜中层厚度的相关性分析) [D] Dalian Medical University. (大连医学大学) 2013.
**A77**	Ma Xiangguo, Zhao Linying, Chang Jie, et al. (马相国, 赵林英, 常婕, 等) An investigation of carotid atherosclerosis in Elunchun ethnicity by ultrasonography. (超声检查颈动脉对鄂伦春民族动脉粥样硬化的调查分析) [J] Yiayao Qianyan. (医药前沿) 2013(17):30-31.
**A78**	Qian Jiajia. (钱佳佳) Research on atherosclerotic characteristics in carotid arteries and its related factors in people aged 20-59 years. (20-59 岁人群颈动脉超声影像学特征及其相关因素的研究) [D] Nanjing Normal University. (南京师范大学) 2013.
**A79**	Zhou Guirong, Liu Aiping. (周桂荣, 刘爱萍) An epidemiological investigation on carotid atherosclerotic plaque in rural dwellers in Mentougou district, Beijing. (北京市门头沟区农村居民颈动脉粥样硬化流行病学调查) [J] Journal of Hebei Medical University. (河北医科大学学报) 2013, 34(10):1212-1212.
**A80**	Liu Beibei, Hua Yang, Jia Lingyun, et al. (刘蓓蓓, 华扬, 贾凌云, 等) Carotid atherosclerotic lesions detected with ultrasonography in the population ≥55 years of age in Beijing area. (北京部分社区 55 岁及以上人群颈动脉粥样硬化性病变的超声筛查) [J] Chinese Journal of Cerebrovascular Diseases. (中国脑血管病杂志) 2011, 08(8):397-401.
**A81**	Sun Jing, Wu Qiang, Fan Chunzhi, et al. (孙静, 武强, 范春芝, 等) Evaluation of the atherosclerotic characteristics in carotid arteries and lower extremity arteries by ultrasonography in individuals of Wanshoulu community in Beijing. (超声对北京万寿路社区老年人群颈动脉和下肢动脉粥样硬化调查分析) [J] Chinese Journal of Medical Ultrasound (Electronic Edition). (中华医学超声杂志(电子版)) 2011, 08(12).
**A82**	Yajuan Hu, Yanhui Liu, Ning Wang. (胡亚娟, 刘燕辉, 王宁) Prevalence of carotid atherosclerosis in an urban district of Beijing and its risk factors. (儿童期肥胖及高血压预测成年期颈动脉粥样硬化的前瞻性研究) [J] Chinese General Practice. (中国全科医学) 2012, 15(23):2688-2690.

Notes: The Chinese publication list uses the official English names or abbreviations of the journals. English titles were obtained from the journals themselves or from literature databases (China National Knowledge Infrastructure, Wanfang, and China Science and Technology Journal Database). If an official English translation of a journal name is not available, a Pinyin title is used. If an English translation of a title is not available, we have translated the title, marked it with an asterisk (*), and highlighted it in green.
